# The effect of metformin on ameliorating neurological function deficits and tissue damage in rats following spinal cord injury: A systematic review and network meta-analysis

**DOI:** 10.3389/fnins.2022.946879

**Published:** 2022-08-11

**Authors:** Long-yun Zhou, Xu-qing Chen, Bin-bin Yu, Meng-xiao Pan, Lu Fang, Jian Li, Xue-jun Cui, Min Yao, Xiao Lu

**Affiliations:** ^1^Department of Rehabilitation Medicine, The First Affiliated Hospital of Nanjing Medical University, Nanjing, China; ^2^Department of Otolaryngology, Jiangsu Province Hospital of Chinese Medicine, Affiliated Hospital of Nanjing University of Chinese Medicine, Nanjing, China; ^3^Spine Disease Institute, Longhua Hospital, Shanghai University of Traditional Chinese Medicine, Shanghai, China; ^4^Key Laboratory of Theory and Therapy of Muscles and Bones, Ministry of Education, Shanghai University of Traditional Chinese Medicine, Shanghai, China

**Keywords:** metformin, spinal cord injury, systematic review, neurological function, safety, action mechanism, clinical translation

## Abstract

Spinal cord injury (SCI) is a devastating condition with few treatment options. Metformin, a classical antidiabetic and antioxidant, has extended its application to experimental SCI treatment. Here, we performed a systematic review to evaluate the neurobiological roles of metformin for treating SCI in rats, and to assess the potential for clinical translation. PubMed, Embase, China National Knowledge Infrastructure, WanFang data, SinoMed, and Vip Journal Integration Platform databases were searched from their inception dates to October 2021. Two reviewers independently selected controlled studies evaluating the neurobiological roles of metformin in rats following SCI, extracted data, and assessed the quality of methodology and evidence. Pairwise meta-analyses, subgroup analyses and network analysis were performed to assess the roles of metformin in neurological function and tissue damage in SCI rats. Twelve articles were included in this systematic review. Most of them were of moderate-to-high methodological quality, while the quality of evidence from those studies was not high. Generally, Basso, Beattie, and Bresnahan scores were increased in rats treated with metformin compared with controls, and the weighted mean differences (WMDs) between metformin and control groups exhibited a gradual upward trend from the 3rd (nine studies, *n* = 164, WMD = 0.42, 95% CI = −0.01 to 0.85, *P* = 0.06) to the 28th day after treatment (nine studies, *n* = 136, WMD = 3.48, 95% CI = 2.04 to 4.92, *P* < 0.00001). Metformin intervention was associated with improved inclined plane scores, tissue preservation ratio and number of anterior horn motor neurons. Subgroup analyses indicated an association between neuroprotection and metformin dose. Network meta-analysis showed that 50 mg/kg metformin exhibited greater protection than 10 and 100 mg/kg metformin. The action mechanisms behind metformin were associated with activating adenosine monophosphate-activated protein kinase signaling, regulating mitochondrial function and relieving endoplasmic reticulum stress. Collectively, this review indicates that metformin has a protective effect on SCI with satisfactory safety and we demonstrate a rational mechanism of action; therefore, metformin is a promising candidate for future clinical trials. However, given the limitations of animal experimental methodological and evidence quality, the findings of this pre-clinical review should be interpreted with caution.

## Introduction

Spinal cord injury (SCI) is a catastrophic event associated with high morbidity and mortality (Lucchesi et al., 2019). Most of the SCIs arise from physical trauma, with an incidence of 210 cases and a prevalence of 5,410 cases per million in the United States (Feigin et al., 2021). Worldwide, there are over 700,000 new cases of traumatic SCI annually and ~27.04 million people are affected by SCI, which equates to 9.5 million years of life lived with disability (Kumar et al., 2018; Gbd, 2019; Lucchesi et al., 2019). However, effective therapeutic strategies for this condition remain limited.

The pathological progression of SCI is multifactorial. Contusion or transection is frequently the initial insult, which leads to structural disruption and breakdown of tissue homeostasis promptly (Anjum et al., 2020; Lin et al., 2022). Subsequently, the acute phase of secondary damage, involving oxidative stress, membrane and ionic dysregulation, neurotransmitter toxicity, and vascular dysfunction, glial cell activation, and neuronal death, is triggered (Tran et al., 2018; Anjum et al., 2020; Xu et al., 2020). Despite the wide range of mechanisms involved in these pathological processes, oxidative stress, and adenosine monophosphate-activated protein kinase (AMPK) are believed to be critical for progression of SCI (Visavadiya et al., 2016; Wang et al., 2018). For instance, increased generation of reactive oxygen species (ROS) following central nervous system (CNS) injury can activate mitogen-activated protein kinase (MAPK) or the nod-like receptor family, pyrin domain-containing three (NLRP3) inflammasome to induce an neuroinflammation response, and may also lead to neuronal damage *via* a wide range of mechanisms (Sarkar et al., 2017; Khatri et al., 2018). Then, activation of AMPK signaling can downregulate the formation of ROS, thereby exerting anti-inflammatory and neuroprotective effects on CNS injury (Jiang et al., 2020; Hu et al., 2021).

Metformin is a classical antidiabetic drug that can activate AMPK signaling and reduce ROS production (Apostolova et al., 2020). With a satisfactory safety profile, metformin has been approved by the Food and Drug Administration as a conventional treatment for type 2 diabetes. Recently, metformin has shown promise in the treatment of obesity, cancer, stroke and cardiovascular disease (Luo et al., 2019; Ma et al., 2020). Given the important role of AMPK signaling and excessive ROS production in SCI, metformin is trying to find its new application in flied of SCI (Wang et al., 2020; Zhou et al., 2020a). Multiple pre-clinical studies have reported favorable neuroprotection by metformin in the treatment of SCI (Wang et al., 2020; Zhang et al., 2020). While, Lin et al. (2015) demonstrated a negative result of metformin for SCI. The issues regarding the candidate administration does, time window of intervention and administration times of metformin for SCI remain poorly understood. Additionally, the systematic knowledges of the safety and pharmacological mechanism of metformin in treating SCI are still limited.

Comprehensively understanding of efficacy, administration details, safety, and pharmacological mechanism is critical for evaluating clinical translation. Chen et al. (2021) conducted a systematic review including seven studies to indicate the effects of metformin on locomotor function recovery in rats after SCI, hinting an application prospective of metformin in SCI. Here, we performed an updated and combined review for analyzing all of those parameters. Compared with Chen et al. (2021), we identified 12 references with inconsistent result tendencies to conducted the systematic review. For the parameter of efficacy, we primarily focused on the dynamic changes of locomotor function after metformin intervention, and paid attention to the effects of metformin on the tissue damage in SCI. In regard to administration details, series of variables concerning metformin dose, intervention timing and administration times participate in subgroup analyses. And a network meta-analysis was carried out for predicting the candidate administration does. Furthermore, we combined the advantages of a traditional review for the knowledges about the safety, pharmacokinetics in the CNS, and mechanism in treating SCI of metformin. Under the comprehensive analysis, this review was aimed to adequately evaluate the neurobiological roles of metformin for treating SCI in rats and describe the potential for future clinical trials and applications.

## Materials and methods

### Literature search

PubMed, Embase, China National Knowledge Infrastructure, WanFang data, Vip Journal Integration Platform, and SinoMed databases were searched from their inception dates to October 2021. According to the guide of search strategy (Leenaars et al., 2012), “spinal cord injuries,” “spinal cord injury,” “spinal cord diseases,” “spinal cord compression,” “spinal cord trauma,” “metformin,” “biguanides,” “guanidines,” “rats,” and “murinae” were used as the key search terms. Reference lists of selected articles were also searched to find additional studies. Appendix 1 contains the search strategy in PubMed database.

### Selection of studies

According to the eligibility criteria, two reviewers independently selected the articles by screening the abstracts and full texts. Disagreements were resolved by consensus following discussion with a third reviewer.

### Eligibility criteria

#### Types of study

Controlled studies evaluating the neurobiological roles of metformin in rats following SCI were searched. All clinical case reports or *in vitro* studies were excluded. No language, publication date, or publication status were restricted (Yao et al., 2015).

#### Types of participants

Laboratory rats of any age, sex, or strain that were subjected to SCI induced by compression or contusion were included. Studies with local or global ischemia, traumatic root avulsion, chronic SCI or genetically modified models were excluded (Oliveri et al., 2014; Sng et al., 2022). Additionally, laceration/transection types of SCI were also excluded because these models do not represent the typical crush injury mechanism in humans (Dietz and Curt, 2006; Oliveri et al., 2014).

#### Types of intervention

Any type of metformin intervention compared with a placebo control was included. Placebo controls included saline, vehicle, dimethyl sulfoxide, or no treatment (Tian et al., 2020). Multiple treatment combinations (e.g., metformin plus methylprednisolone) were excluded.

#### Types of outcome measure

The 21-point Basso, Beattie, and Bresnahan (BBB) locomotor rating scale is a widely used ordinal scale involving assessments of hindlimb joints, paw placement during stepping, weight support, and forelimb-hindlimb coordination of animals (Basso et al., 1995). Briefly, examiners subjectively registered the limb movements and walking characteristics in an open-field arena, then assigned a BBB scale score ranging from 0 (complete paralysis) to 21 (normal locomotion) for indicating the basic locomotion of an animal (Zhou et al., 2020b). Only data at the same time points were used in the analyses of BBB scores.

The inclined plane test describes the maximal angle at which rats can maintain themselves on an inclined plane measured in 0–90° (Rivlin and Tator, 1978), and is used to evaluate limb muscle strength. Animals were placed transversely on the rubber inclined plane and the highest angle the rat could maintain for 5 s was recorded (Chen et al., 2018).

In histopathological analysis, tissue preservation area and the number of motor neurons in anterior horn area of the spinal cord are widely applied indicators for injury severity and the prospect of locomotor recovery after SCI (Li et al., 2017; Wang et al., 2020). To reduce the risk of data heterogeneity, only data obtained by the same detection method was used in the analyses.

### Data extraction

Data including the name of the first author, publication year, animal strain and sex, animal age and weight, number of animals, SCI model, spinal level of trauma, metformin administration, and measured outcomes were extracted from included studies. Moreover, to investigate the potential therapeutic mechanism of metformin for SCI, the proposed mechanisms and changes of related molecules were also extracted from those studies. In studies with multiple intervention arms, only data from the metformin and negative control groups were used in our analysis. If data were not numerically described in the text, we estimated the values from graphs using GetData Graph Digitizer 2.26 (http://getdata-graph-digitizer.com; Yao et al., 2015). If data were missing, authors were contacted.

### Risk of bias assessment

The SYstematic Review Centre for Laboratory animal Experimentation risk of bias (SYRCLE's RoB) tool, a specially designed system for animal studies, was employed to assess the methodological quality of the studies (Hooijmans et al., 2014). This tool encompasses 10 items for evaluating selection bias, performance bias, detection bias, attrition bias, reporting bias, and other biases. The items were scored with “yes,” “no,” or “unclear,” indicating a low risk, a high risk or insufficient details to assess the risk of bias, respectively. Two investigators independently assessed the methodological quality according to this tool.

### Assessment of quality of evidence

The assessment of quality of evidence from included studies was based on the previously reported strategy (Charalambous et al., 2014, 2016). This strategy classifies the experimental laboratory animal studies (ELAS) into three groups. The first group includes blinded randomized ELAS (bRELAS) and non-blinded RELAS (nbRELAS). Non-randomized RELAS (nRELAS) and uncontrolled ELAS (UELAS) were defined as the second group. Case series and reports belong to the third group. The studies in the first group were considered to provide higher quality evidence, followed by the studies in the second and third group. Additionally, a three-part system of evidence quality assessment was used to indicate the strengths and weaknesses of each study within each group, including study group sizes, subject enrolment quality and overall risk of bias based on SYRCLE's RoB tool. For example, bRELAS with large group sizes, clear inclusion criteria, thorough diagnostic investigations and low overall risk of bias were considered to provide the highest available quality of evidence (Charalambous et al., 2016).

### Statistical analyses

Pairwise meta-analyses and subgroup analyses were produced with Review Manager version 5.3 (provided by the Cochrane Collaboration). For all outcome measures, differences were considered statistically significant at *P* < 0.05 (two-tailed). Pooled data for each outcome were reported as weighted mean differences (WMDs) with 95% confidence intervals (CIs). Heterogeneity between studies was evaluated by χ^2^ and Cochrane's *I*^2^ (Higgins and Green, 2011). The random effect model was applied because it provides the expected average of all samples of individual true effect sizes, rather than a single common true effect size from dispersed studies caused by sampling errors. Line graphs were constructed by GraphPad Prism 8.0.2 software (GraphPad Software, San Diego, California USA) to highlight the dynamic WMDs of BBB scores and dynamic BBB score improvements in both groups (Sng et al., 2022).

To explore the appropriate dose of metformin, a network meta-analysis was preformed according to the Bayesian method using Stata 12.0 (StataCorp LP, College Station, Texas, USA) to simultaneously compare direct and indirect treatment regimens. Probability of being the best intervention and surface under cumulative ranking (SUCRA) are usually applied to determine the rank order of interventions (Mbuagbaw et al., 2017; Owen et al., 2020). Stata reports both the probabilities and SUCRA based on the reported computational formula (Chiocchia et al., 2020). For every treatment dose, we calculated the probabilities of its efficacy, and then ranked the treatments according to SUCRA.

## Results

### Study selection

**Figure 1:**
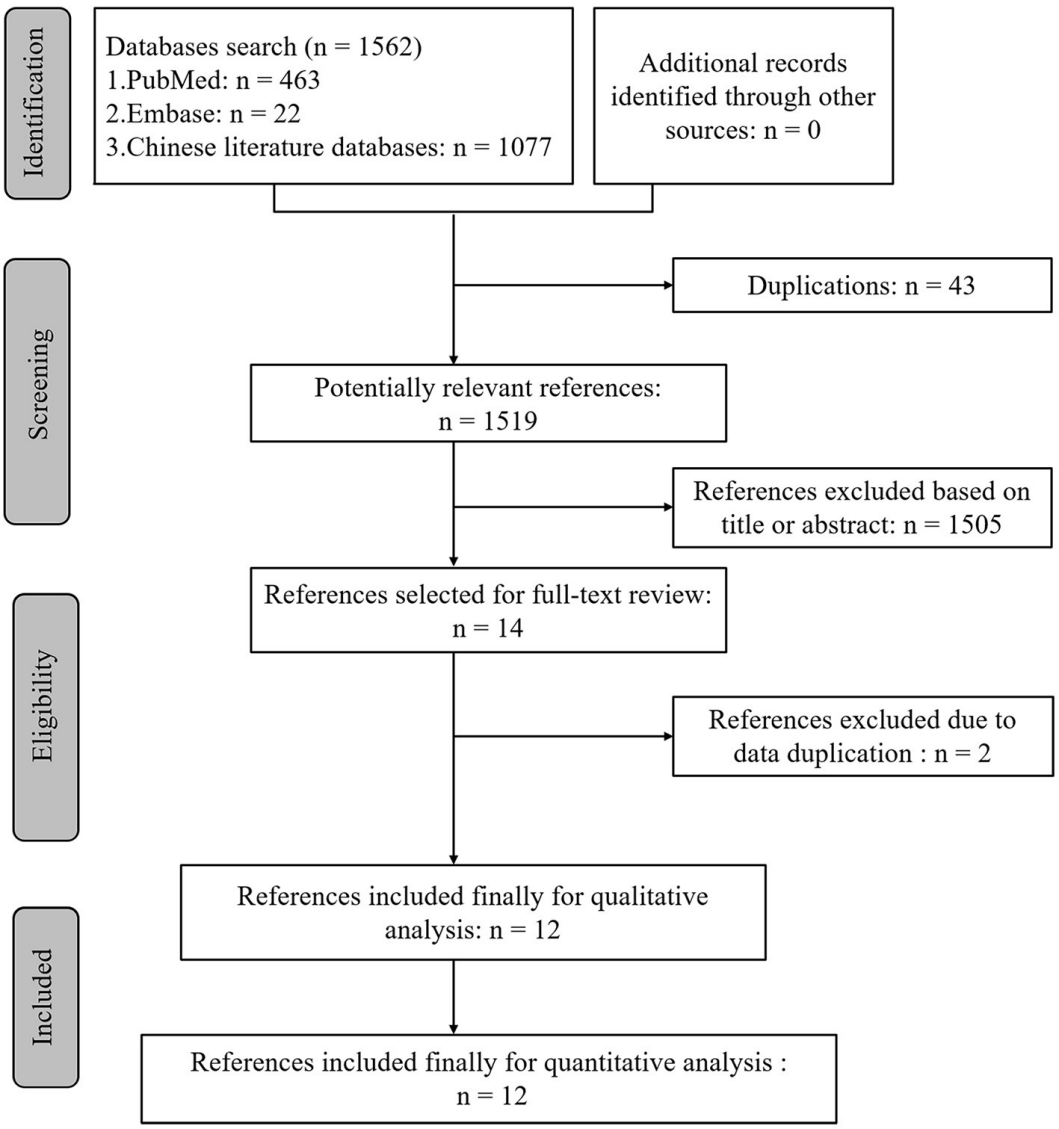
Summary of the literature identification and selection process.

Our systematic search identified 1,562 potentially relevant references. Following the removal of duplications and title/abstract screening, 14 papers were selected for full-text screening. Two publications (Zhang, 2017; Zhao, 2021) were excluded at the full-text stage because the data in these articles were reported in Zhang (2017) and Zhao (2021), respectively. Twelve articles were, therefore, included in this systematic review and meta-analysis (Figure 1; Lin et al., 2015; Wang et al., 2016, 2018, 2020; Zhang et al., 2017a,b, 2020; Afshari et al., 2018; Guo et al., 2018, 2019; Wu et al., 2021; Zhao et al., 2021).

### Characteristics of the included studies

Of the 12 publications from 2015 to 2021, 10 were presented in English, the other two in Chinese (Guo et al., 2019; Zhao et al., 2021). The sample size ranged from 18 to 114 animals. Seven publications demonstrated an SCI model induced by aneurysm clip compression, and the others used a weight-drop impactor method (Wang et al., 2016; Guo et al., 2018, 2019; Zhang et al., 2020; Zhao et al., 2021). All studies caused trauma at the T7–T11 level. Eight studies reported the source of metformin (Lin et al., 2015; Wang et al., 2016, 2018; Zhang et al., 2017a,b; Afshari et al., 2018; Guo et al., 2019; Wu et al., 2021). The placebo controls included saline and dimethyl sulfoxide diluted with saline. Metformin dose ranged from 10 to 320 mg/kg, and all studies used intraperitoneal injection except Lin et al. (2015). Most studies injected metformin immediately post injury, while five studies treated SCI from the 1st day or 1st week after trauma (Lin et al., 2015; Wang et al., 2016; Guo et al., 2018; Zhang et al., 2020; Zhao et al., 2021). Ten studies applied repeated administration of metformin, once daily for 3–28 days after SCI, whereas a single dose of metformin was employed in the remaining studies (Table 1; Afshari et al., 2018; Wang et al., 2018).

**Table 1:** Characteristics of studies included in the meta-analysis.

**References**	**Animals**	**Injury model**	**Animal number**	**Metformin source**	**Experimental groups**	**Control group**	**Outcome**
Wu et al. (2021)	108 female SD rats (220–250 g)	T9 vascular clip compression 15 g * 1 min	36/36/36	Sigma	A: SCI + metformin (50 mg/kg i.p., immediately post injury and once daily until sacrifice)	B: sham SCI + saline C: SCI + saline	Behavioral: BBB scale, ICP, footprint analysis Histopathology: HE staining, LFB staining, Nissl staining, immunohistochemistry. IF Other: Western blot, PCR
Zhao et al. (2021)	104 male SD rats (250–300 g)	T9-11 weight-drop impactor 10 g * 60 mm	17/17/17/17; 12/12/12	N/A	1A: SCI + metformin (200 mg/kg i.p., immediately post injury and once daily until sacrifice); 2A: SCI + metformin (200 mg/kg i.p., immediately post injury and once daily until sacrifice);	1B: sham SCI + saline 1C: sham SCI + metformin 1D: SCI + saline; 2B: SCI + saline 2C: SCI + metformin + SC-1 (10 μl intrathecal injection, 30 min piror to injury)	Behavioral: BBB scale, ICP Histopathology: TTC staining, IF Other: Western blot, PCR, dot blot
Wang et al. (2020)	70 female SD rats (220–250 g)	T9 vascular Clip compression 15 g* 1 min	10/10/10; 10/10/10/10	N/A	1A: SCI + metformin (50 mg/kg i.p., immediately post injury and once daily for 14 d); 2A: SCI + metformin (50 mg/kg i.p., immediately post injury and once daily for 14 d)	1B: sham SCI 1C: SCI + saline; 2B: sham SCI 2C: SCI + saline 2D: SCI + metformin + LY294002 (1.2 mg/kg i.p., immediately post injury and once daily for 14 d)	Behavioral: BBB scale, footprint analysis Histopathology: HE staining, Nissl staining, TUNEL, IF Other: Western blot
Zhang et al. (2020)	90 male SD rats (8–12 weeks, 180–220 g)	T9-10 weight-drop impactor 10 g * 30 mm	18/18/18/18/18	N/A	A: SCI + metformin (50 mg/kg i.p., once daily for 14 d post injury)	B: sham SCI + saline C: SCI + saline D: SCI + metformin + XAV939 (0.4 mg/kg i.p., once daily for 14 d post injury) E: SCI + MP (30 mg/kg i.p., immediately once daily for 14 d post injury)	Behavioral: BBB scale Histopathology: HE staining, Nissl staining, TUNEL, IF Other: Western blot
Guo et al. (2019)	36 female SD rats (250–300 g)	T9-T11 weight-drop impactor	12/12/12	MedChem Express	A: SCI + metformin (50 mg/kg i.p., immediately post injury and once daily for 7 d)	B: sham SCI C: SCI + saline	Behavioral: BBB scale Histopathology: TUNEL, IF Other: Western blot, PCR
Afshari et al. (2018)	48 male SD rats (240–260 g)	T9 aneurysmal Clip compression 110 g* 1 min	8/8/8/8/8/8	Sigma	A: SCI + metformin (10 mg/kg i.p., immediately post injury) B: SCI + metformin (50 mg/kg i.p., immediately post injury) C: SCI + metformin (100 mg/kg i.p., immediately post injury)	D: sham SCI + saline E: SCI + saline F: SCI + minocycline (90 mg/kg i.p., immediately post injury and 45 mg/kg i.p., q12 h for 1 d)	Behavioral: BBB scale, tail-flick latency, von Frey filaments test Histopathology: HE staining Other: Body weight, ELISA
Guo et al. (2018)	60 female SD rats (200–240 g)	T9-10 weight-drop impactor 10 g * 50 mm	20/20/20	N/A	A: SCI + metformin (10 mg/kg i.p., once daily for 3 d post injury)	B: sham SCI + saline C: SCI + saline	Behavioral: BBB scale Histopathology: Nissl staining, IF Other: Western blot
Wang et al. (2018)	50 male SD rats (200–220 g)	T7-T10 bulldog clamp compression 30 g * 1 min	10/10/10/10/10	Boyun Biotechnology	A: SCI + metformin (50 mg/kg i.p., immediately and 24 h post injury)	B: SCI + saline C: SCI + compound C (20 mg/kg immediately and 24 h post injury) D: SCI + EPO (2,000 U/kg immediately and 24 h post injury) E: SCI + EPO + compound C	Behavioral: BBB scale, ICP Histopathology: Nissl staining Other: Western blot
Zhang et al. (2017a)	114 adult female SD rats (220–250 g)	T9 level vascular clip compression 30 g * 1 min	18/18/18; 15/15/15/15	MedChem Express	1A: SCI + metformin (50 mg/kg i.p., immediately post injury and once daily for 28 d); 2A: SCI + metformin (50 mg/kg i.p., immediately post injury and once daily for 28 d)	1B: Sham group 1C: SCI + saline; 2B: Sham group 2C: SCI + saline 2D: SCI + metformin + chloroquine (50 mg/kg i.p., immediately post injury and once daily for 28 d post injury)	Behavioral: BBB scale, ICP Histopathology: HE staining, Nissl staining, IF Other: Western blot, transmission electron microscopy
Zhang et al. (2017b)	105 adult female SD rats (220–250 g)	T9 level vascular clip compression 30 g * 1 min	35/35/35	MedChem Express	A: SCI + metformin (50 mg/kg i.p., immediately post injury and once daily for 14 d)	B: Sham group C: SCI + saline	Behavioral: BBB scale Histopathology: HE staining, IF Other: Western blot, PCR, gelatin zymography, fluorimetric assay of MMP-9, myeloperoxidase activity, Evans blue, spinal cord oedema
Wang et al. (2016)	48 adult female SD rats (180–220 g)	T9-10 weight-drop impactor 10 g * 25 mm	14/6/14/14	Sigma Aldrich	A: SCI + metformin (200 mg/kg i.p., once daily for 14 d prior to injury) B: SCI + metformin (100 mg/kg i.p., once daily for 3 d post injury)	C: Sham group D: SCI + vehicle	Behavioral: BBB scale Histopathology: HE staining, Nissl staining IF Other: Western blot
Lin et al. (2015)	18 male Wistar rats	T8-9 level aneurysm Clip compression 55 g * 1 min	6/6/6	Tocris Bioscience	A: SCI + metformin (320 mg/kg i.v., once daily for 20 d after 1 week of SCI)	B: Sham group C: SCI + vehicle (DMSO diluted with saline)	Behavioral: BBB scale, ICP, limb hanging test, pain test Other: Western blot

*BBB, Basso, Beattie, and Bresnahan; DMSO, dimethyl sulfoxide; ELISA, enzyme-linked immunosorbent assay; HE, hematoxylin-eosin; IF, immunofluorescence; ICP, inclined plane test; i.p., intraperitoneal injection; LFB, luxol fast blue; MMP-9, matrix metalloproteinase-9; MP, methylprednisolone; N/A, not available; PCR, Polymerase chain reaction; SD, Sprague-Dawley; SCI, spinal cord injury; TTC, 3,5-TriphenylTetrazolium chloride; TUNEL, Terminal dexynucleotidyl transferase mediated d UTP nick end labeling*.

### Risk of bias within studies

The mean number of reported items in SYRCLE's RoB tools was 4.5. Six studies (50%) adequately described 5–6 items in the standard checklist list (Lin et al., 2015; Zhang et al., 2017a,b, 2020; Afshari et al., 2018; Wu et al., 2021). The items of “baseline characteristics,” “selective outcome reporting,” and “outcome assessor blinding” were well-evaluated items, with an appropriate description in 100, 100, and 91.7% of included studies, respectively. In contrast, the items of “sequence generation,” “allocation concealment,” “random housing,” “investigator blinding,” and “random outcome assessment” were poorly delineated in included studies, since none or few of them reported those details. Incomplete outcome data was adequately addressed in four studies (Lin et al., 2015; Zhang et al., 2017a,b; Wu et al., 2021). In additional, we did not identify any other sources of bias such as pooling of drugs, dropouts, unit of analysis error, design-specific bias, and bias due to inappropriate influence of funder (Table 2).

**Table 2:** Risk of bias summary.

**Study**	**1**	**2**	**3**	**4**	**5**	**6**	**7**	**8**	**9**	**10**
Wu et al. (2021)	Unclear	Yes	Unclear	Unclear	Unclear	Unclear	Yes	Yes	Yes	Yes
Zhao et al. (2021)	Unclear	Yes	Unclear	Unclear	Unclear	Unclear	Yes	Unclear	Yes	Yes
Wang et al. (2020)	Unclear	Yes	Unclear	Unclear	Unclear	Unclear	Yes	Unclear	Yes	Yes
Zhang et al. (2020)	Yes	Yes	Unclear	Unclear	Unclear	Yes	Yes	Unclear	Yes	Yes
Guo et al. (2019)	Unclear	Yes	Unclear	Unclear	Unclear	Unclear	Unclear	Unclear	Yes	Yes
Afshari et al. (2018)	Unclear	Yes	Unclear	Unclear	Yes	Unclear	Yes	Unclear	Yes	Yes
Guo et al. (2018)	Unclear	Yes	Unclear	Unclear	Unclear	Unclear	Yes	Unclear	Yes	Yes
Wang et al. (2018)	Unclear	Yes	Unclear	Unclear	Unclear	Unclear	Yes	Unclear	Yes	Yes
Zhang et al. (2017a)	Unclear	Yes	Unclear	Unclear	Unclear	Unclear	Yes	Yes	Yes	Yes
Zhang et al. (2017b)	Unclear	Yes	Unclear	Unclear	Unclear	Unclear	Yes	Yes	Yes	Yes
Wang et al. (2016)	Unclear	Yes	Unclear	Unclear	Unclear	Unclear	Yes	Unclear	Yes	Yes
Lin et al. (2015)	Unclear	Yes	Unclear	Unclear	Unclear	Unclear	Yes	Yes	Yes	Yes

“*Yes” indicates low risk of bias; “No” indicates high risk of bias; “Unclear” represents unclear risk of bias. This tool includes 10 questions: (1) sequence generation; (2) baseline characteristics; (3) allocation concealment; (4) random housing; (5) investigator blinding; (6) random outcome assessment; (7) outcome assessor blinding; (8) incomplete outcome data; (9) selective outcome reporting; (10) other sources of bias*.

### Quality of evidence within studies

Overall, the majority of the studies included in this review did not offer high quality of evidence (Table 3). Half of included studies were classified into bRELAS or nbRELAS (Wang et al., 2016; Guo et al., 2018, 2019; Zhang et al., 2020; Wu et al., 2021; Zhao et al., 2021), the remaining belonged to the nRELAS. In addition, five study included a small study population size (Zhang et al., 2017a,b; Guo et al., 2018; Wang et al., 2018; Zhao et al., 2021). Only three studies adequately addressed details of the subject enrolment quality (Zhang et al., 2020; Wu et al., 2021; Zhao et al., 2021). A potential risk of bias was widely presented in methodological quality of included studies. Thus, the results based on those studies should be interpreted with caution.

**Table 3:** Summaries of the quality of evidence of included studies.

**References**	**Study design**	**Study group sizes**	**Subject enrolment quality**	**Overall risk of bias**
Wu et al. (2021)	bRELAS	Moderate	Fairly	Low/Moderate
Zhao et al. (2021)	bRELAS	Small	Fairly	Moderate
Wang et al. (2020)	nRELAS	Good	Unclear	Moderate
Zhang et al. (2020)	bRELAS	Moderate	Fairly	Low/Moderate
Guo et al. (2019)	nbRELAS	Moderate	Unclear	Moderate/High
Afshari et al. (2018)	nRELAS	Moderate	Unclear	Low/Moderate
Guo et al. (2018)	bRELAS	Small	Unclear	Moderate
Wang et al. (2018)	nRELAS	Small	Unclear	Moderate
Zhang et al. (2017a)	nRELAS	Small	Unclear	Low/Moderate
Zhang et al. (2017b)	nRELAS	Small	Unclear	Low/Moderate
Wang et al. (2016)	bRELAS	Moderate	Unclear	Moderate
Lin et al. (2015)	nRELAS	Moderate	Unclear	Low/Moderate

*bRELAS, blinded randomized experimental laboratory animal studies; nbRELAS, non-blinded randomized experimental laboratory animal studies; nRELAS, non-randomized experimental laboratory animal studies*.

### Overall analysis of the effects of metformin

**Figure 2:**
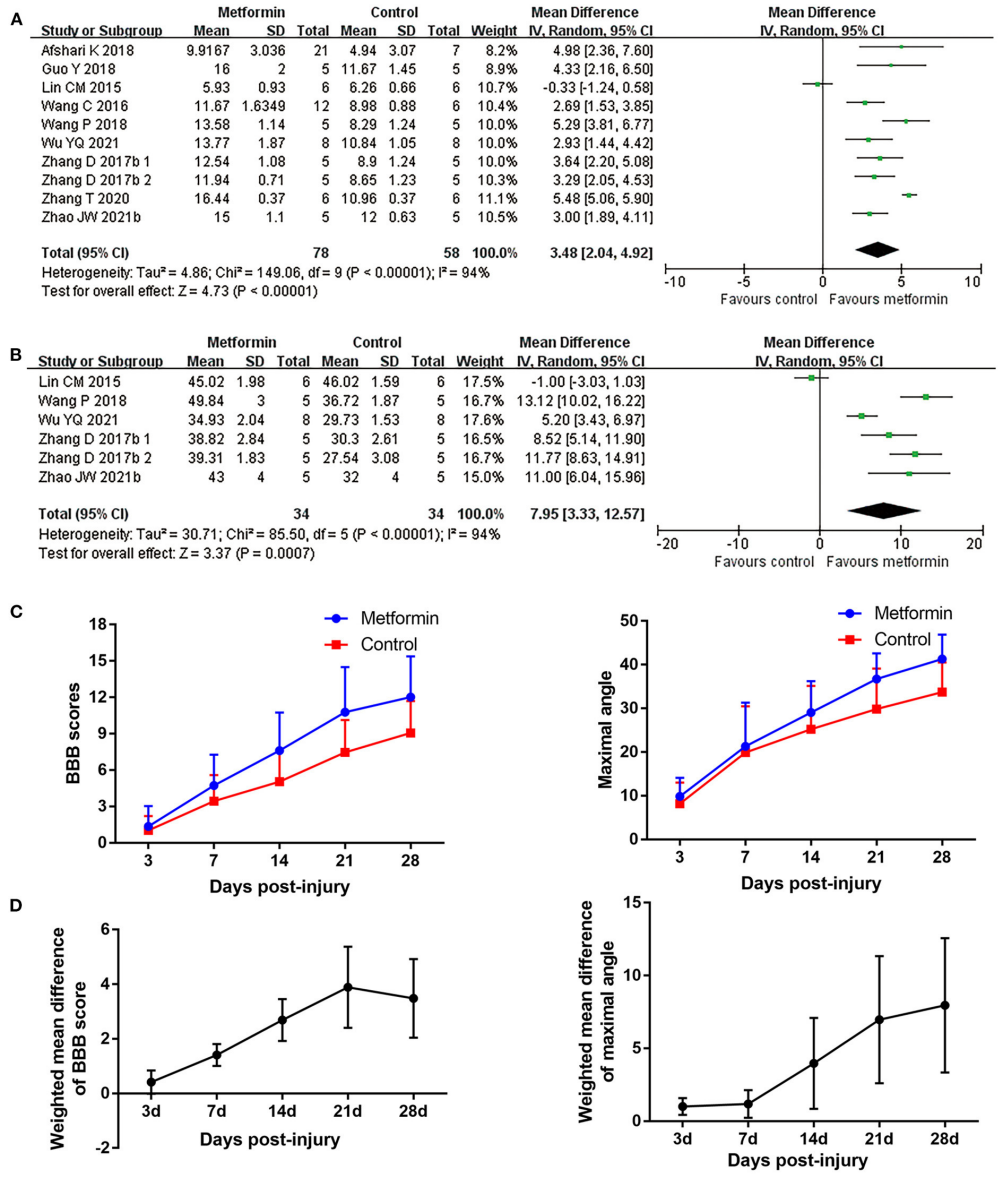
Overall analyses of the effects of metformin on dynamic changes of neurological function. **(A,B)** BBB scale, inclined plane test meta-analysis at 28th day after SCI. **(C)** The BBB scores and inclined plane scores in each group over time. **(D)** The WMDs of BBB score between metformin and control groups from 3rd to 28th day after SCI.

BBB scores was employed in all included studies to evaluate locomotor recovery in rats. Nine studies collected dynamic BBB score information from 1 to 28 days after SCI (Lin et al., 2015; Wang et al., 2016, 2018; Zhang et al., 2017a, 2020; Afshari et al., 2018; Guo et al., 2018; Wu et al., 2021; Zhao et al., 2021). All studies except one (Lin et al., 2015) reported improved BBB scores in metformin-treated rats compared with placebo intervention. The meta-analyses and dynamic BBB scores in both groups also revealed that BBB scores were increased in metformin-treated animals compared with controls over the 7th (12 studies, *n* = 198, WMD = 1.41, 95% CI = 1.01–1.80, *P* < 0.00001) to 28th day after injury (nine studies, *n* = 136, WMD = 3.48, 95% CI = 2.04–4.92, *P* < 0.00001), and the WMDs between the two groups showed a gradual upward trend with time (Figures 2A,C,D and Table 4). Furthermore, meta-analyses regarding inclined plane scores confirmed the positive recovery effects of metformin for rats subjected to SCI (Figure 2B and Table 4).

**Table 4:** Summary of overall analyses of the effects of metformin.

**Outcome title**	**No. of studies**	**No. of animals**	**Weighted mean difference**		**Heterogeneity**	
			**95% CI**	***P*-value**	***I* ^2^**	***P*-value**
1 BBB scores	12	198				
1.1 BBB scale at day 3	9	164	0.42 [−0.01, 0.85]	0.06	72	0.0004
1.2 BBB scale at day 7	12	198	1.41 [1.01, 1.80]	<0.00001	52	0.01
1.3 BBB scale at day 14	11	186	2.69 [1.92, 3.47]	<0.00001	82	<0.00001
1.4 BBB scale at day 21	8	120	3.89 [2.50, 5.28]	<0.00001	91	<0.00001
1.5 BBB scale at day 28	9	136	3.48 [2.04, 4.92]	<0.00001	94	<0.00001
2 Inclined plane test	5	68				
2.1 Inclined plane test at day 3	4	46	1.00 [0.42, 1.58]	0.0008	0	0.63
2.2 Inclined plane test at day 7	5	68	1.18 [0.23, 2.12]	0.01	48	0.08
2.3 Inclined plane test at day 14	5	68	3.97 [0.84, 7.09]	0.01	88	<0.00001
2.4 Inclined plane test at day 21	4	52	6.97 [2.61, 11.33]	0.002	90	<0.00001
2.5 Inclined plane test at day 28	5	68	7.95 [3.33, 12.57]	0.0007	94	<0.00001
3 Tissue preservation area	4	54	13.29 [2.66, 23.93]	0.01	98	<0.00001
4 Number of anterior horn motor neurons	4	32	14.71 [3.30, 26.12]	0.01	97	<0.00001

**Figure 3:**
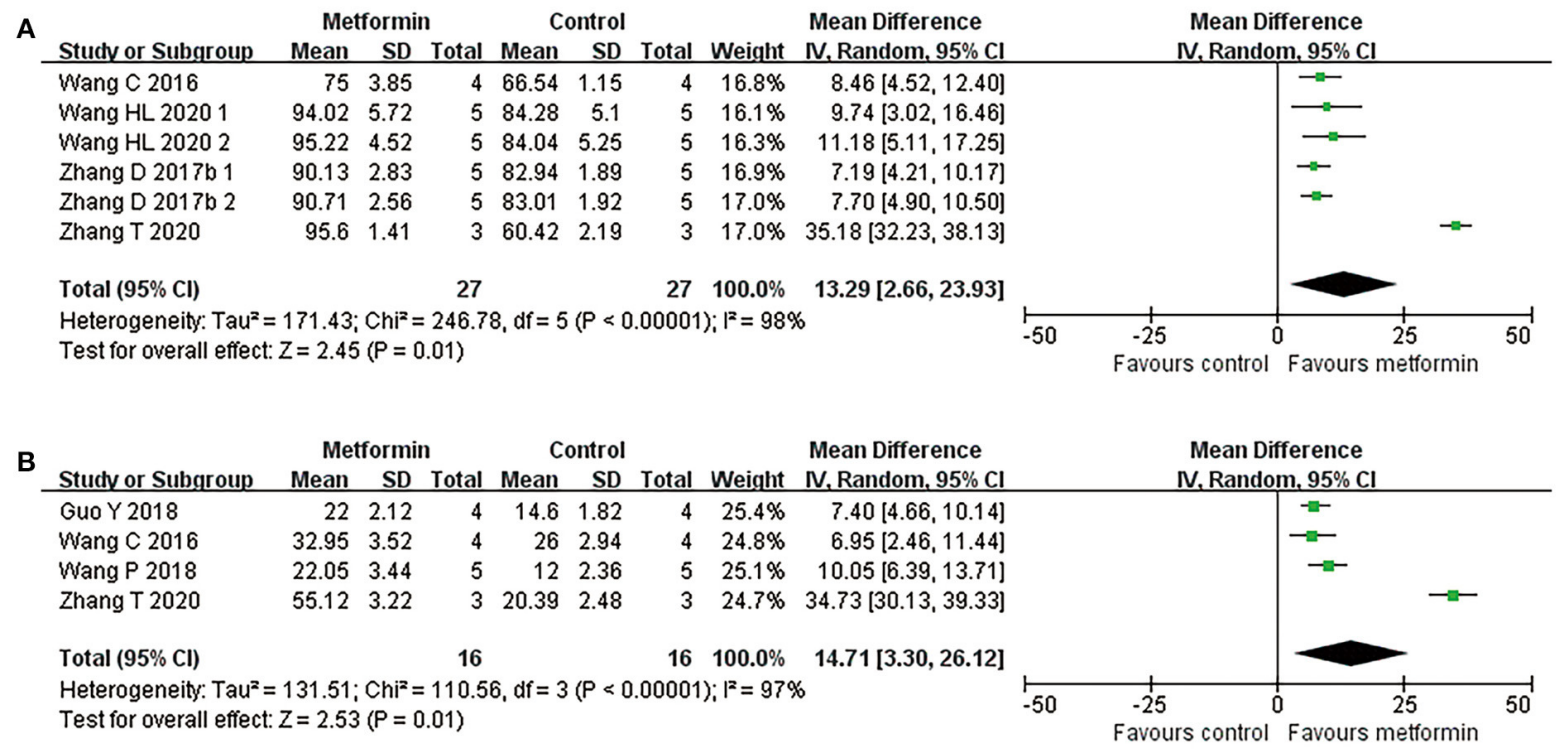
Overall analyses of the effects of metformin on tissue damage in lesion area. **(A,B)** Tissue preservation area ratio, number of survival motor neuron meta-analysis in lesion area and the effect sizes line chart.

Four studies applied hematoxylin-eosin staining to describe the preservation area in the form residual tissue/cross-sectional area ratio (Wang et al., 2016, 2020; Zhang et al., 2017a, 2020). Meta-analyses revealed metformin treatment resulted in a higher percentage of preserved tissue compared with controls (four studies, *n* = 54, WMD = 13.29, 95% CI = 2.66–23.93, *P* = 0.01, Figure 3 and Table 4). Additionally, another study with lesion area data in longitudinal sections demonstrated suppression of lesion area in the injury site after metformin treatment (Wu et al., 2021).

By counting the Nissl positive cells in anterior horn area of the spinal cord, six publications reported the number of motor neurons in this area (Wang et al., 2016, 2018; Zhang et al., 2017a, 2020; Guo et al., 2018; Wu et al., 2021). Of the five studies reporting the mean number of anterior horn motor neuron in a single cross section, one presented these data in the epicenter and in both side-regions of the injury separately (Zhang et al., 2017a), while the remaining studies calculated this mean count in the global lesion site (Wang et al., 2016, 2018; Guo et al., 2018; Zhang et al., 2020). Consistently, data presented in different injury segments or meta-analyses of the four studies both suggested an increased number of survived motor neurons in metformin-treated rats compared with controls (four studies, *n* = 32, WMD = 14.71, 95% CI =3.30–26.12, *P* = 0.01, Figure 3 and Table 4).

### Subgroup analyses of the effects of metformin

**Figure 4:**
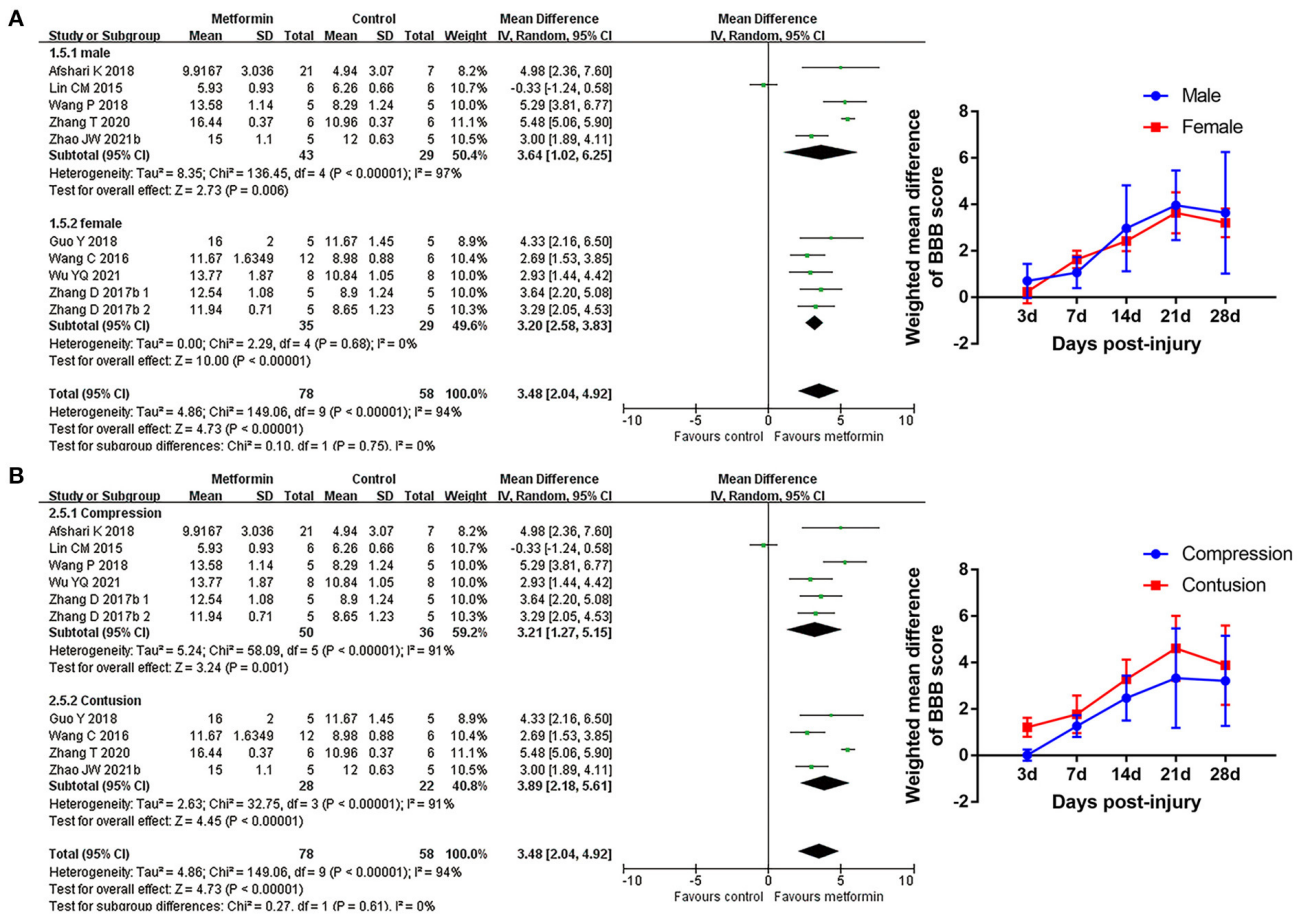
BBB scale subgroup analysis concerning rat gender and injury model type. **(A,B)** Subgroup analysis concerning rat gender and injury model type at 28th day after SCI and the WMDs of BBB score over time in different subgroups.

**Figure 5:**
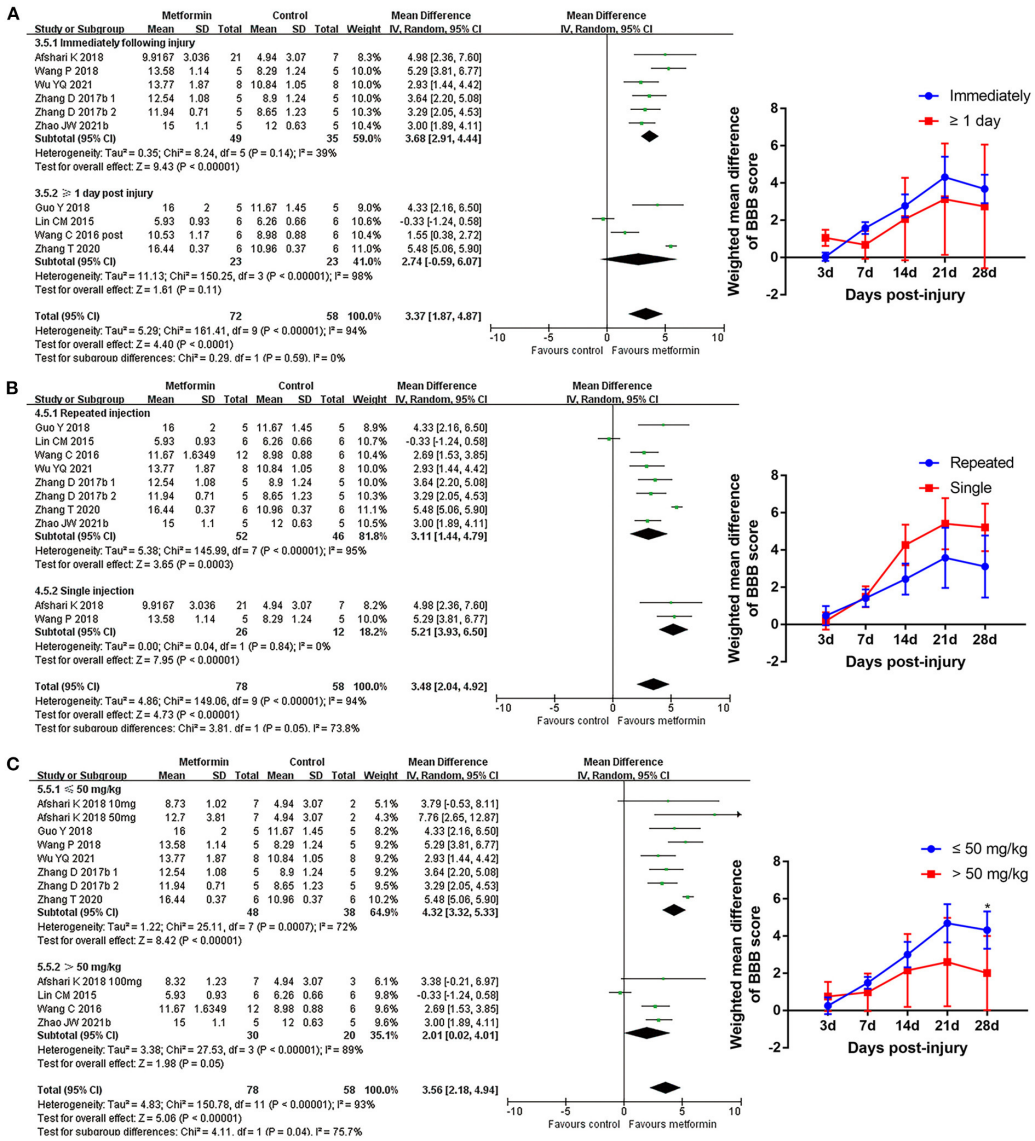
BBB scale subgroup analysis concerning administration details. **(A–C)** Subgroup analysis concerning administration timing, injection numbers and administration dose at 28th day after SCI and the WMDs of BBB score over time in different subgroups. *p < 0.05.

Subgroup analyses indicated that there was no statistical difference in BBB scores between rats based on gender, compression, or contusion injury model, timing of metformin administration (immediately or from the 1st day following injury), or number of injections Figures 4, 5, Supplementary Figure 1, and Table 5). Dose was argued to critically impact metformin efficacy (Riddle, 2000; Araújo et al., 2017). Interestingly, analyses with respect to metformin doses indicated an association of locomotor recovery with administration dose (Figure 5 and Table 5). Recent studies suggest that locomotor function, systemic symptoms, and pathology may differ depending on the level of SCI and rat strain used (Wilcox et al., 2017; Hong et al., 2018). However, analysis of distinct strains and injury levels was not preformed because these factors were consistent in our included studies.

**Table 5:** Subgroup analyses of the effects of metformin.

**Subgroup title**	**No. of studies**	**No. of animals**	**Weighted mean difference**		**Heterogeneity**		**Subgroup difference**
			**95% CI**	***P*-value**	***I* ^2^**	***P*-value**	
1 Rat gender	12	198					
1.1 BBB scale at 3rd day	9	164					*P* = 0.31
1.1.1 Male	3	50	0.70 [−0.04, 1.45]	0.06	75	0.02	
1.1.2 Female	6	114	0.24 [−0.26, 0.74]	0.35	64	0.02	
1.2 BBB scale at 7th day	12	198					*P* = 0.14
1.2.1 Male	5	72	1.06 [0.39, 1.72]	0.002	69	0.01	
1.2.2 Female	7	126	1.63 [1.25, 2.01]	<0.00001	0	0.44	
1.3 BBB scale at 14th day	11	186					*P* = 0.57
1.3.1 Male	5	72	2.97 [1.12, 4.83]	0.002	93	<0.00001	
1.3.2 Female	6	114	2.42 [1.99, 2.85]	<0.00001	0	0.44	
1.4 BBB scale at 21th day	8	120					*P* = 0.81
1.4.1 Male	5	72	3.96 [1.46, 6.47]	0.002	95	<0.00001	
1.4.2 Female	3	48	3.64 [2.75, 4.54]	<0.00001	48	0.12	
1.5 BBB scale at 28th day	9	136					*P* = 0.75
1.5.1 Male	5	72	3.61 [1.02, 6.25]	0.006	97	<0.00001	
1.5.2 Female	4	64	3.20 [2.58, 3.83]	<0.00001	0	0.68	
2 Injury model	12	198					
2.1 BBB scale at 3rd day	9	164					*P* < 0.00001
2.1.1 Compression	5	114	0.01 [−0.23, 0.25]	0.93	0	0.58	
2.1.2 Contusion	4	50	1.21 [0.80, 1.62]	<0.00001	0	0.73	
2.2 BBB scale at 7th day	12	198					*P* = 0.29
2.2.1 Compression	7	136	1.26 [0.79, 1.72]	<0.00001	51	<0.00001	
2.2.2 Contusion	5	62	1.77 [0.96, 2.57]	<0.0001	59	0.05	
2.3 BBB scale at 14th day	11	186					*P* = 0.21
2.3.1 Compression	7	136	2.47 [1.50, 3.43]	<0.00001	81	<0.00001	
2.3.2 Contusion	4	50	3.28 [2.43, 4.13]	<0.00001	55	0.08	
2.4 BBB scale at 21th day	8	120					*P* = 0.33
2.4.1 Compression	4	70	3.33 [1.19, 5.47]	0.002	93	<0.00001	
2.4.2 Contusion	4	50	4.61 [3.21, 6.01]	<0.00001	77	0.004	
2.5 BBB scale at 28th day	9	136					*P* = 0.61
2.5.1 Compression	5	86	3.21 [1.27, 5.15]	0.001	91	<0.00001	
2.5.2 Contusion	4	50	3.89 [2.18, 5.61]	<0.00001	91	<0.00001	
3 Administration timing	12	192					
3.1 BBB scale at 3rd day	9	158					*P* < 0.0001
3.1.1 Immediately	6	124	0.04 [−0.19, 0.27]	0.71	0	0.50	
3.1.2 ≥ 1 day	3	34	1.05 [0.61, 1.50]	<0.00001	3	0.36	
3.2 BBB scale at 7th day	12	192					*P* = 0.03
3.2.1 Immediately	8	146	1.58 [1.26, 1.90]	<0.00001	0	0.56	
3.2.2 ≥ 1 day	4	46	0.68 [−0.07, 1.42]	0.07	57	0.07	
3.3 BBB scale at 14th da	11	180					*P* = 0.54
3.3.1 Immediately	8	134	2.77 [2.15, 3.39]	<0.00001	49	0.05	
3.3.2 ≥ 1 day	4	46	2.06 [−0.16, 4.28]	0.07	95	<0.00001	
3.4 BBB scale at 21th day	8	114					*P* = 0.47
3.4.1 Immediately	4	68	4.31 [3.21, 5.49]	<0.00001	67	0.02	
3.4.2 ≥ 1 day	4	46	3.13 [0.14, 6.12]	0.04	97	<0.00001	
3.5 BBB scale at 28th day	9	130					*P* = 0.59
3.5.1 Immediately	5	84	3.68 [2.91, 4.44]	<0.00001	39	0.14	
3.5.2 ≥ 1 day	4	46	2.74 [−0.59, 6.07]	0.11	98	<0.00001	
4 Number of injections	12	198					
4.1 BBB scale at 3rd day	9	164					*P* = 0.44
4.1.1 Repeated injection	8	136	0.47 [−0.05, 0.98]	0.07	75	0.0002	
4.1.2 Single injection	1	28	0.19 [−0.28, 0.67]	0.42			
4.2 BBB scale at 7th day	12	198					*P* = 0.83
4.2.1 Repeated injection	10	160	1.41 [0.94, 1.88]	<0.00001	56	0.006	
4.2.2 Single injection	2	38	1.49 [0.93, 2.05]	<0.00001	0	0.73	
4.3 BBB scale at 14th day	11	186					*P* = 0.009
4.3.1 Repeated injection	9	148	2.44 [1.60, 3.28]	<0.00001	84	<0.00001	
4.3.2 Single injection	2	38	4.27 [3.18, 5.36]	<0.00001	0	0.84	
4.4 BBB scale at 21th day	8	120					*P* = 0.09
4.4.1 Repeated injection	6	82	3.58 [1.96, 5.20]	<0.0001	93	<0.00001	
4.4.2 Single injection	2	38	5.41 [4.03, 6.80]	<0.00001	23	0.25	
4.5 BBB scale at 28th day	9	136					*P* = 0.05
4.5.1 Repeated injection	7	98	3.11 [1.44, 4.79]	0.0003	95	<0.00001	
4.5.2 Single injection	2	38	5.21 [3.93, 6.50]	<0.00001	0	0.84	
5 Administration dose	12	198					
5.1 BBB scale at 3rd day	9	164					*P* = 0.28
5.1.1 ≤ 50 mg/kg	7	126	0.25 [−0.20, 0.70]	0.27	70	0.001	
5.1.2 > 50 mg/kg	3	38	0.75 [−0.04, 1.54]	0.06	57	0.10	
5.2 BBB scale at 7th day	12	198					*P* = 0.34
5.2.1 ≤ 50 mg/kg	9	148	1.49 [1.17, 1.81]	<0.00001	10	0.001	
5.2.2 > 50 mg/kg	4	50	0.98 [−0.03, 1.99]	0.06	66	0.03	
5.3 BBB scale at 14th day	11	186					*P* = 0.42
5.3.1 ≤ 50 mg/kg	8	136	3.00 [2.31, 3.69]	<0.00001	67	0.0007	
5.3.2 > 50 mg/kg	4	50	2.15 [0.19, 4.11]	0.03	86	<0.0001	
5.4 BBB scale at 21th day	8	120					*P* = 0.11
5.4.1 ≤ 50 mg/kg	5	70	4.69 [3.66, 5.72]	<0.00001	73	0.001	
5.4.2 > 50 mg/kg	4	50	2.60 [0.22, 4.98]	0.03	88	<0.0001	
5.5 BBB scale at 28th day	9	136					*P* = 0.04
5.5.1 ≤ 50 mg/kg	6	86	4.32 [3.32, 5.33]	<0.00001	72	0.0007	
5.5.2 > 50 mg/kg	4	50	2.01 [0.02, 4.01]	0.05	89	<0.00001	

### Network analysis of the effects of metformin dose

**Figure 6:**
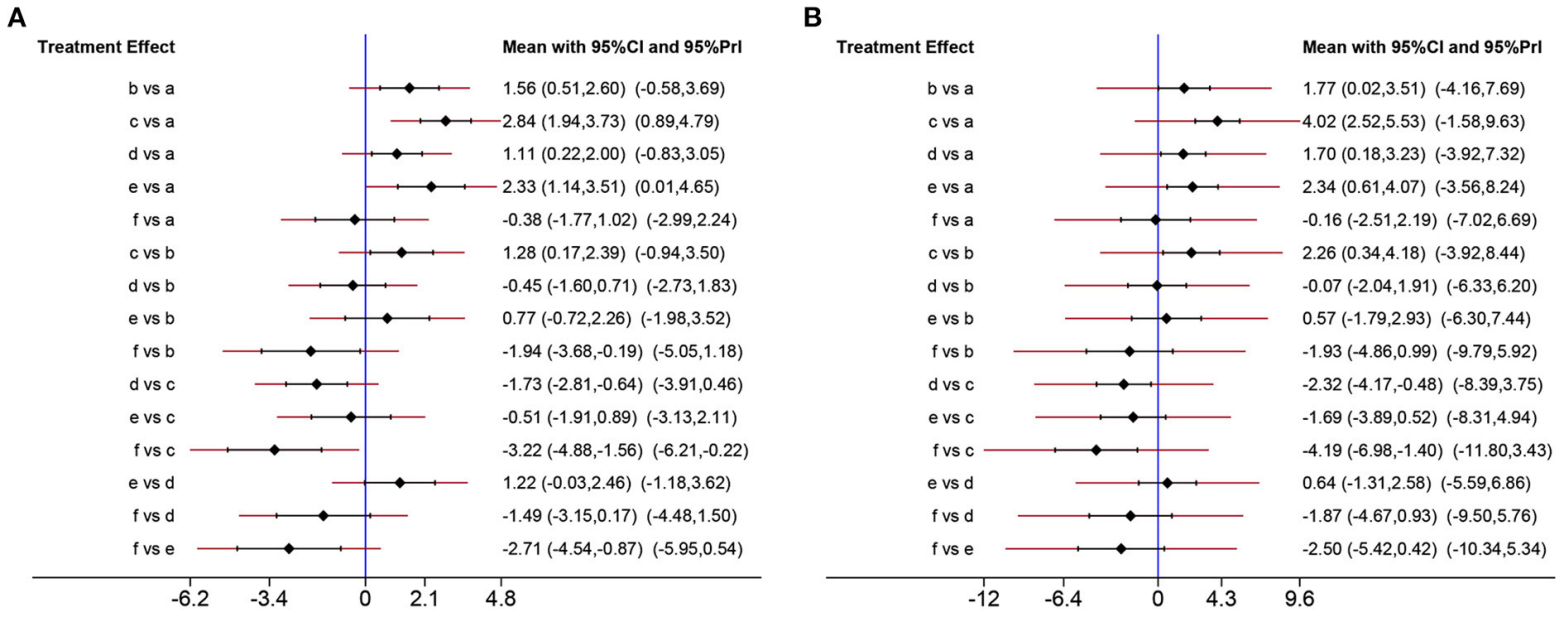
Network analysis of the effects of metformin at different doses. **(A)** Forest plot of effect size in different metformin doses according to data on 28th day. **(B)** Forest plot of effect size in different metformin doses according to data on 21th day. a, Control; b, 10 mg/kg metformin; c, 50 mg/kg metformin; d, 100 mg/kg metformin; e, 200 mg/kg metformin; f, 320 mg/kg metformin.

**Figure 7:**
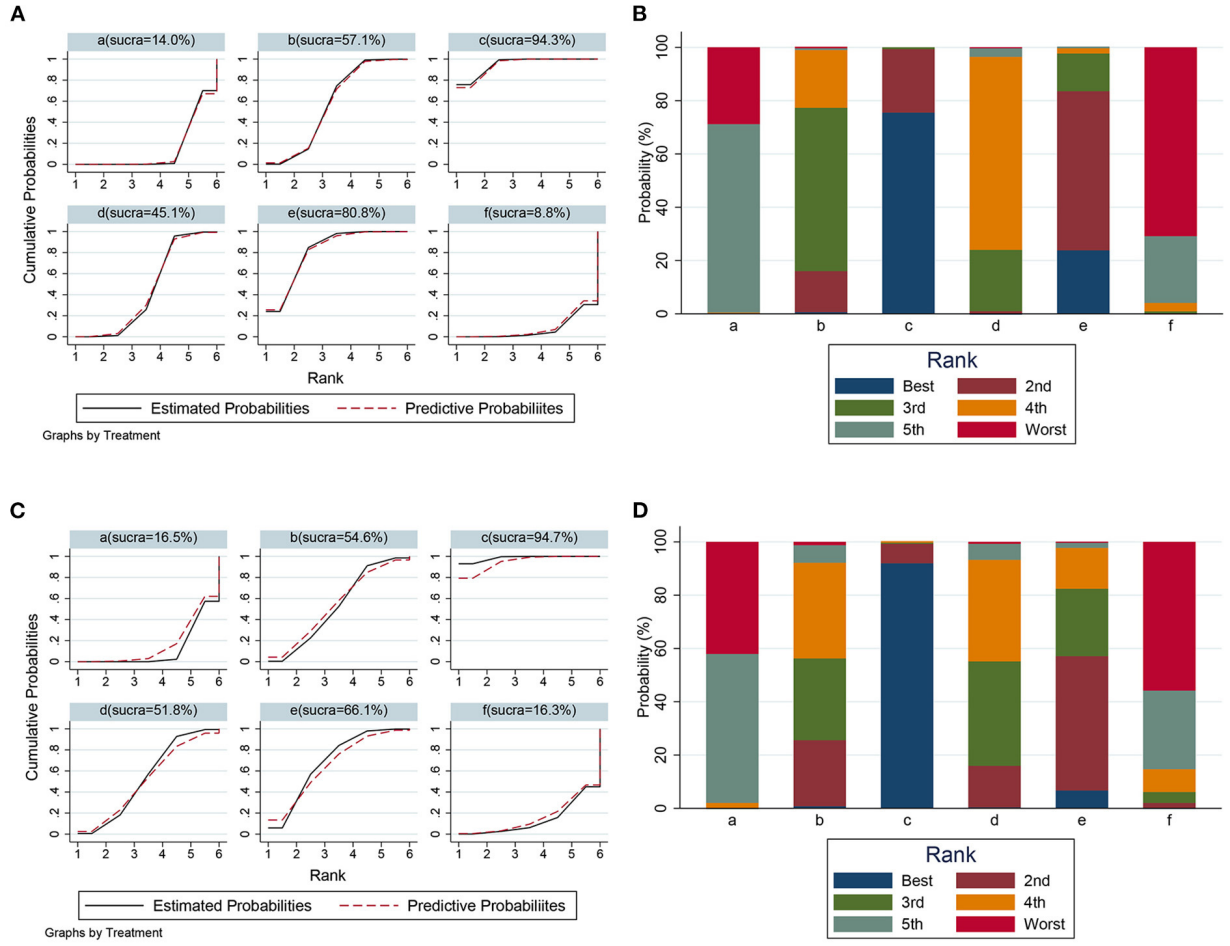
The SUCRA value and probabilities of each treatment doses. **(A)** SUCRA value ranking of different administration doses according to data on 28th day. **(B)** Histogram of ranking probability of each treatment dose according to data on 28th day. **(C)** SUCRA value ranking of different administration doses according to data on 21th day. **(D)** Histogram of ranking probability of each treatment dose according to data on 21th day. a, control; b, 10 mg/kg metformin; c, 50 mg/kg metformin; d, 100 mg/kg metformin, e, 200 mg/kg metformin; f, 320 mg/kg metformin.

To comprehensively compare the effects of metformin at different doses, BBB scores from day 14 to 28 after SCI were employed in our network analysis. A forest plot using data from the 28th day revealed that 50 mg/kg metformin exhibited a greater effect compared with 10, 100, and 200 mg/kg metformin (Figure 6), and seemed to be the best treatment regimen on the basis of the SUCRA value (Figures 7A,B). The superior treatment effect and SUCRA value for 50 mg/kg metformin was confirmed on the 14th and 21st days after SCI (Figures 7C,D, Supplementary Figure 2).

### Sensitivity analysis

We performed sensitivity analyses by excluding either non-randomized studies, small-sample-sized studies, or all single studies. Through heterogeneity among studies were partly reduced by excluding specific studies, a moderate or high heterogeneity remained. Moreover, the improvement of BBB scores with metformin was largely maintained (Supplementary Table 1), indicating a robust result concerning BBB scores.

### Proposed therapeutic mechanisms

The proposed mechanisms behind metformin in included studies was presented in Table 6. Generally, activating adenosine monophosphate-activated protein kinase signaling, regulating mitochondrial function and relieving endoplasmic reticulum stress were showed to be highly related to the action mechanisms underlying the neuroprotective effects of metformin for experimental SCI.

**Table 6:** The proposed mechanism of the protective effect of metformin for SCI.

**References**	**Proposed mechanism**	**Effects**
Wu et al. (2021)	Regulation of AMPK and mTOR signaling to enhance autophagy	Increased p-AMPK, ATG7, LC3-II and LAMP1, and decreased p-mTOR and p62
Zhao et al. (2021)	Activation of TET2-FoxO3a axis	Increased TET2, enhanced interaction between TET2 and Foxo3a
Wang et al. (2020)	1. Regulation of mitochondrial dysfunction and oxidative stress 2. stabilizing microtubule	1. Increased p-AKT, Nrf2, HO-1, NQO1 and mitochondrial membrane potential, inhibited ROS 2. Increased Ace-tubulin and MAP2, and decreased Tyr-tubulin
Zhang et al. (2020)	Regulation of Wnt/β-catenin signaling	Increased β-catenin
Guo et al. (2019)	Alleviation of endoplasmic reticulum stress	Decreased GRP78, CHOP, caspase-12, and cleaved caspase-3
Afshari et al. (2018)	Inhibition of neuroinflammation	Decreased TNF-α and IL-1β
Guo et al. (2018)	Inhibition of mTOR signaling to enhance autophagy	Decreased mTOR, p70S6K and p62, increased Beclin1, and LC3-II/ I ratio
Wang et al. (2018)	Regulation of AMPK and mTOR signaling	Increased p-AMPK, LC3-II/I ratio and Beclin 1, decreased p-mTOR, p-p70S6K, and p62
Zhang et al. (2017a)	Regulation of AMPK and mTOR signaling to enhance autophagy	Increased p-AMPK, LC3-II and Beclin 1, decreased p-mTOR, p-p70S6K and p62
Zhang et al. (2017b)	1. Inhibiting neutrophil infiltration 2. Regulation of AMPK signaling	1. Decreased ICAM-1 and MMP-9 2. Increased p-AMPK
Wang et al. (2016)	1. Regulation of mTOR signaling to enhance autophagy 2. Suppressing neuroinflammation and apoptosis	1. Decreased p-mTOR, p-p70S6K, increased LC3B-II and Beclin 1 2. Decreased NF-κB, cleaved caspase-3, increased Bcl-2
Lin et al. (2015)	Regulation of AMPK signaling	Increased p-AMPK

*Ace-tubulin, acetylated tubulin; AKT, protein kinase B; ATG7, autophagy related protein 7; Bcl-2, B cell lymphoma-2; CHOP, CCAAT/enhancer-binding protein homologous protein; FoxO3a, forkhead box O3a; GRP78, glucose-regulated protein 78; HO-1, heme oxygenase-1; IL-1β, interleukin-1 beta; LC3-II, light chain 3-II; LAMP1, Lysosomal-associated membrane protein 1; MAP2, microtubule-associated protein-2; MMP-9, matrixmetalloproteinase-9; mTOR, mammalian target of rapamycin; NF-κB, nuclear factor-kappaB; NQO1, NAD(P)H: quinone oxidoreductase 1; Nrf2, nuclear factor (erythroid-derived 2)-like; ROS, reactive oxygen species; TET2, Ten-eleven translocation-2; TNF-α, tumor necrosis factor-α; Tyr-tubulin, tyrosinated tubulin; p-AMPK, phosphorylated adenosine monophosphate-activated protein kinase; p70S6K, p70 ribosomal protein S6 kinase*.

## Discussion

Treatments for SCI have been a research focus for a long time; however, this has produced few effective therapies. Therefore, it is of great importance that clinical translation of novel treatments is achieved. For translation to the clinic, treatments must be efficacious, safe with minor adverse effects, and a clear molecular mechanism need to be determined (Park et al., 2017). Metformin is a promising candidate for SCI treatment; therefore, we conducted a systematic review of all available studies to evaluate its potential for clinical translation.

### Summary of evidence

This review identified 12 eligible studies that compared metformin with placebo controls in SCI models. Pairwise meta-analyses indicated increased BBB scores in the metformin group, and this effect tended to increase over time. There were no differences in locomotor recovery with respect to gender, compression or contusion injury, administration timing of metformin or number of injections; however, there were differences related to the administration dose. Network meta-analysis confirmed the candidate dose with the best neuroprotective effects.

Meta-analysis also revealed that the WMDs of the maximal angle maintained on the inclined plane showed a gradually increasing trend (from 1.00° on the 3rd day to 7.95° on the 28th day) between metformin and control groups. Compared with controls, metformin-treated animals exhibited a higher percentage of preserved tissue at injury sites. Additionally, an increased number of motor neurons in anterior horn area of the spinal cord was observed in metformin-treated rats vs. controls.

The proposed mechanisms in included studies implied that metformin could modulate the AMPK and mTORC1 signaling, ameliorate endoplasmic reticulum stress and regulate the oxidative stress, thereby exerting a satisfactory neuroprotective role in SCI rat.

The quality of included studies was the basis for the meta-analysis. Although most of included studies had moderate-to-high methodological quality, the quality of evidence from those studies was not high. The above results need to be interpreted with caution given those limitations.

### Statistical heterogeneity

Substantial between-study heterogeneity on treatment effect were observed in our analyses, which may become the barrier to conduct the meaningful meta-analysis and conclude the correct decision. There are several variables concerning used animal, injury type, injury degree, administration details, random performance, and result tendency. In addition, though only data at the same time points were used in the analyses for BBB scores, the understanding of assessor for this indicator varies from each other. All of those factors may contribute to the heterogeneity among studies. Here, subgroup analyses and sensitive analyses targeted potential factors were employed for answering the heterogeneous reasons. In our study, subgroup analyses concerning variables rat gender, administration dose or number of injections all reduced the result heterogeneity to a certain degree. The Cochrane's *I*^2^ in subgroup of single injection ranged from 0 to 23%, and subgroup analyses regarding different metformin doses regulated the heterogeneities in both subgroups. Lin et al. (2015) showed a negative effect of metformin for SCI, which may be related to the application of metformin at a high dose. Our sensitivity analyses from day 7 to 28 after SCI stably suggested a minimum heterogeneity after excluding Lin et al. (2015) compared with excluding other studies. Importantly, SYRCLE's RoB tools revealed a potential bias in methodological quality, especially in the design of random and allocation concealment. Those bias may contribute to a heterogeneous classification between the studies and lead to the treatment effect heterogeneity. In summary, multiple variables among included studies may be associated with the presented heterogeneity; different administration details, distinct results, and varied methodological quality of studies appear to be the important factors increasing the heterogeneity in our meta-analyses. The subgroup analysis under subgroups to control multiple variables is the feasible measure for reducing heterogeneity and explore the heterogeneity sources, while this type of analysis is difficult to be conducted in our study due to the insufficiency in included studies.

### Metformin dose

The efficacy of metformin decreases at both very low and high doses (Riddle, 2000). One included study reported that injured rats administrated with 320 mg/kg metformin did not show improvement in motor function or neuropathic pain (Lin et al., 2015). Afshari et al. (2018) indicated that metformin at 50 mg/kg had the highest effect on promoting locomotor recovery and alleviating SCI complications compared with 10 and 100 mg/kg doses. Another study showed higher efficacy in attenuating the inflammatory response with a metformin dose of 50 mg/kg vs. high doses of 100 and 200 mg/kg (Araújo et al., 2017). Consistently, our subgroup analyses demonstrated a superior locomotor recovery in administration of metformin at ≤ 50 mg/kg. Further network meta-analysis suggested that 50 mg/kg metformin may provide better neuroprotection than other doses of metformin. Equivalent dose calculation indicates that 50 mg/kg metformin in rodents is below the therapeutic dose used for diabetes treatment in humans (Sanchez-Rangel and Inzucchi, 2017), indicating that dose titration of metformin needs to be considered for human SCI trials.

The decreased efficacy of metformin at high doses on SCI may be related to its role on metabolism. Metformin does not significantly impact the plasma glucose levels in healthy participants (Sambol et al., 1996). However, a rise in postprandial plasma glucose or insulin resistance were observed in SCI or brain injury, 100 mg/kg metformin obviously lowered the plasma glucose to a physiological level in rats subjected to those diseases (Elder et al., 2004; Zhang et al., 2022). The transport of glucose from the periphery to lesion site is reported to be diminished post-SCI (Jaiswal et al., 2022), thus administration of metformin at dose of ≥100 mg/kg may further deteriorate the glucose uptake impairment in vulnerable lesion site. Given the critical role of glucose metabolism for CNS developing and homeostasis (Veys et al., 2020), this negative impact of high doses of metformin seems be detrimental for recovery of SCI. Secondly, in the internal environment of CNS, metformin was suggested to accelerate consumption of extracellular glucose (Westhaus et al., 2017; Blumrich and Dringen, 2019). While, due to its inhibition role on mitochondrial respiration chain, metformin at a high dose (comparable to 140 mg/kg in rats) strongly elevated glycolysis (Li et al., 2019). Consequently, the ATP synthesis pathway is disrupted and lactate largely accumulates in cells (Blumrich and Dringen, 2019; Li et al., 2019), which is detrimental for neuroregeneration and may cause the further damage of neurol cells in spinal cord (Ohnishi et al., 2021). Collectively, metformin at a high dose may notably impact the glucose uptake of CNS, dimmish the ATP synthesis in CNS and lead to the lactate accumulation in neurol cells, thereby neutralizing its neuroprotective effects. However, the specific dose at which metformin obviously abolishes its neuroprotection in SCI remain obscure and may warrant the further investigation.

### Timing of metformin administration

The progress of pathology following SCI has a distinct timeframe (Tran et al., 2018). Most included studies performed the design of administrating metformin immediately or from the 1st day following SCI. Our subgroup analyses indicated the neurological improvement did not differ between administrating metformin immediately and from the 1st day following SCI. However, due to those reported administration timing all located in the acute phase of SCI, this result does not mean delayed administration shows no impact the efficacy of metformin. Early treatment of secondary damage is critical for neurological recovery in SCI participants (Badhiwala et al., 2019). Consistently, in a study in which metformin was administrated 1 week after SCI, no significant difference in locomotor function was observed between metformin and placebo treatment (Lin et al., 2015). Meanwhile, the action of metformin against SCI was shown to partly depend on its modulation of mTORC1 activation, oxidative stress and the inflammatory response, which is usually triggered a few hours after trauma to the spinal cord (Anjum et al., 2020; Wang et al., 2020). Therefore, early metformin administration needs to be considered although clinical interventions applied within a few hours after SCI are not practical.

### Pharmacokinetics of metformin

Accumulation in the CNS by crossing the blood–brain barrier is powerful evidence for a drug's potential effect on a CNS disease. Pharmacokinetic detection suggested oral administration of metformin can rapidly lead to its accumulation in brain tissues (Hong et al., 2019), and rats under inflammatory conditions exhibited brain region-specific differences in metformin distribution compared with normal rats (łabuzek et al., 2010). Meanwhile, łabuzek et al. (2010) reported a higher metformin concentration in cerebrospinal fluid compared with that in plasma 6 h after administration. The pharmacokinetic parameters of metformin have also been established in healthy, young and old individuals, pregnant women, and in patients with diabetes and brain injury (Graham et al., 2011; Jang et al., 2016). Generally, metformin pharmacokinetics were closely related to the conditions of subjects (Graham et al., 2011). A decrease in maximum concentration and elimination rate constant were shown in traumatic brain injury patients vs. healthy subjects, while the apparent distribution volume and peak time displayed an opposite trend, indicating delayed metformin absorption in traumatic brain injury patients (Taheri et al., 2019). Despite an absence of metformin pharmacokinetic information in SCI, the above findings indicate that monitoring metformin plasma levels may need to be considered in human trials.

### Safety

Metformin is approved by the Food and Drug Administration for type II diabetes mellitus typically, and is exploring new use in the field of obesity, cancer and aging (Ayoub et al., 2020; Samaras et al., 2020; Masarwa et al., 2021). Side effects of metformin are primarily mild-to-moderate digestive disturbance, and rarely hypoglycemia, anemia or lactic acidosis (Mccreight et al., 2016). Although high doses of metformin present some toxic or adverse effects, there is a wide range between the median effective dose and the median lethal dose. For instance, in animal studies, metformin at doses of 10–200 mg/kg exert neuroprotective effects (Afshari et al., 2018; Zhao et al., 2021), even at a dose of 500 mg/kg, which mimics accumulation, the mortality rate in animals with sepsis is not aggravated (Gras et al., 2006). In human trials focused on type II diabetes mellitus, benefits were observed with as little as 500 mg/day, while only mild or moderate adverse effects were reported with the maximum recommended dose of 2,500 mg/day (Garber et al., 1997). Clearance of metformin is mainly implemented in the kidney (Lalau et al., 2018). Despite general avoidance of metformin for stage 4–5 chronic kidney disease, these patients show a relatively low occurrence of lactic acidosis or hypoglycemia, even under repeated administration of a high dose (2,000 mg/day; Tanner et al., 2019). Importantly, no events of hypoglycemia or lactic acidosis occurred in severe traumatic brain injury patients treated with 2,000 mg/day metformin (Taheri et al., 2019). However, the metformin safety specially targeted SCI patients remains to be confirmed due to the lack of related human clinical trials.

### Potential therapeutic mechanisms

According to the proposed mechanisms in included studies and related evidences, we speculated the potential mechanisms of metformin, which are described as follows.

#### Activation of AMPK

AMPK is a well-known cellular energy sensor. Progression of SCI is accompanied by disruption of energy metabolism, excessive oxidative stress and a reactive increase of AMPK (Hu et al., 2021). Further activation of AMPK can effectively alleviate the inflammatory response and neuronal apoptosis in SCI by regulating oxidative stress, autophagy or the family of caspase proteins (Jiang et al., 2020; Zhou et al., 2020a). Metformin has been generally argued to be an activator of AMPK signaling (Apostolova et al., 2020). Interestingly, AMPK is notably activated by metformin treatment in animals with SCI, which subsequently enhances the autophagy and alleviates neuronal damage (Zhang et al., 2017a; Wang et al., 2018; Wu et al., 2021). Thus, based on the critical role of AMPK in CNS diseases, activation of AMPK may be an important mechanism of metformin for ameliorating neuronal damage and neurological function deficits.

#### Inhibition of mTOR complex 1

mTOR complex 1 (mTORC1) is a direct target of AMPK, and plays an important role in anabolic and catabolic processes. Insult to the spinal cord triggers mTORC1 activation in the lesion area (Kjell et al., 2014). The activation of mTORC1 in SCI and other CNS diseases contributes to microglia activation, astrogliosis and neuronal death (Nikolaeva et al., 2016; Lin et al., 2020). Suppression of mTORC1 signaling is a major pathway affected by metformin in the treatment of diabetes, cancer and cardiovascular disease (Efentakis et al., 2019). Importantly, the activity of mTOR and p70S6 kinase, which is a subunit or substrate of mTORC1, is downregulated in a rat model of SCI after metformin intervention (Wang et al., 2016; Zhang et al., 2017a; Guo et al., 2018). Taken together, these findings indicate that the action of metformin against SCI may partly depend on its negative effect on mTORC1.

#### Regulation of mitochondrial function

Mitochondrial disorder can lead to aberrant electron flow and a consequent increase in ROS production (Scialò et al., 2020). The generated ROS further activate the inflammasome and apoptosis signaling to induce neuroinflammation or neural damage (Wu et al., 2020). Numerous studies have suggested a specific inhibitory action of metformin on mitochondrial complex I (Apostolova et al., 2020). Metformin significantly reduces mitochondrial ROS formation by selective inhibition of the reverse electron flow through its mild inhibitory action on complex I (Batandier et al., 2006). Interestingly, improved mitochondrial function and reduced oxidative stress was exhibited in SCI rats after treatment by metformin (Wang et al., 2020), indicating involvement of a mitochondrial mechanism in metformin neuroprotection after SCI.

#### Alleviating endoplasmic reticulum stress

Finally, metformin can ameliorate endoplasmic reticulum stress and the unfolded protein response in SCI through modulation of CCAAT/enhancer-binding protein homologous protein or X-box binding protein 1 signaling (Guo et al., 2019). Suppression of the pathways involved in endoplasmic reticulum stress was suggested to promote neuronal survival in CNS diseases (Zhong et al., 2020; Chuan et al., 2021). Therefore, we speculate that alleviating endoplasmic reticulum stress represents another potential mechanism by which metformin protects the neurons in lesion area.

**Figure 8:**
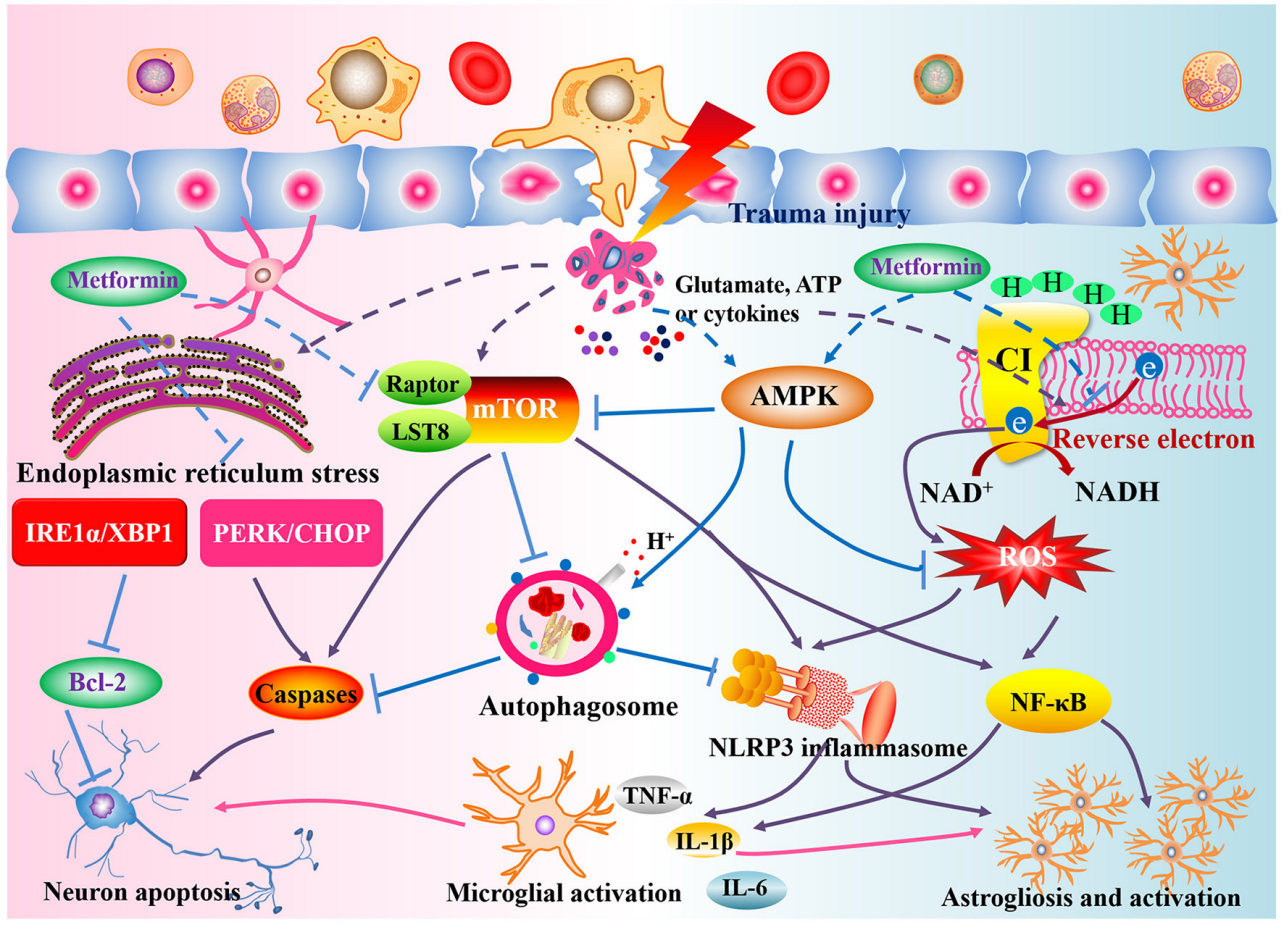
Potential action mechanisms of metformin against SCI. Following the primary insult, structural damage and a loss of homeostasis trigger the disturbance of energy metabolism, oxidative stress, mitochondrial disorder, endoplasmic reticulum stress, and increased mTORC1 expression. Metformin, the activator of AMPK and well-antioxidant, can modulate the AMPK and reverse electron flow in mitochondria to suppress the ROS generation and activation of NLRP3 inflammasome or NF-κB signaling, ameliorate endoplasmic reticulum stress to regulate the Bcl-2 and caspase-3, attenuate mTORC1 activation to induce the autophagosome formation, thereby exerting a satisfactory neuroprotective role in SCI rat.

In addition to the mechanisms mentioned, metformin also acts on TET2- FoxO3a axis, Wnt/β-catenin signaling and neuroinflammation responses (Afshari et al., 2018; Zhang et al., 2020; Zhao et al., 2021). The therapeutic mechanisms by which metformin affects SCI appear to be multiple (Figure 8) and further research will clarify its actions.

### Strengths and limitations

Chen et al. have performed the first meta-analysis to evaluate the efficacy of metformin on rats with SCI, revealing a potential therapeutic method for this disease. While, understanding of efficacy, dose and timing of administration, safety, and pharmacological mechanism is critical for effective clinical translation. Here, we employed a combination of updated systematic and traditional review to comprehensively analyze all of these parameters. The findings indicate that metformin warrants further investigation with respect to SCI intervention. Greater knowledge of metformin will facilitate research into SCI pathology and promote investigation of new drugs to achieve clinical translation.

It is important to note that there are several limitations of our study. Firstly, substantial heterogeneity in treatment effects between the studies was presented in our review. The heterogeneity in our study seems stubborn and complicated, which may be a barrier to meaningful analysis. While, the robust results in the sensitivity analyses, dynamic changes of locomotor function and nearly consistent result tendency among studies enhance the strength of evidence regarding metformin efficacy. Secondly, most of our findings were based on the analysis of BBB scores. This scale is a well-documented tool, while the accuracy of this outcome largely depends on assessments of locomotion that are made subjectively. The possibility of subjective bias in included studies may cause misinterpretation of metformin effects. Thirdly, although we applied an extensive search strategy, the risk of missing potentially relevant articles remains because we did not have access to all possible databases and only searched for studies in English or Chinese. Additionally, subgroup analysis concerning injury level or rat strains was not performed because of the inconsistency of these factors in our included studies.

## Conclusion

In summary, our present review shows that metformin can promote motor recovery and attenuate tissue damage in SCI. Metformin shows satisfactory safety in animal studies of SCI and human trials, with a potential mechanism of action. Hence, we suggest that metformin is suitable for further confirmation in clinical trials, which may result in a new clinical therapy for SCI. Nonetheless, to move toward the clinical trials, extensive pre-clinical studies must be performed to understand the mechanism of metformin. Besides, in light of the limitations of methodological and evidence quality within included studies, the findings of this pre-clinical review should be interpreted with caution.

## Data availability statement

The original contributions presented in the study are included in the article/Supplementary material, further inquiries can be directed to the corresponding author/s.

## Author contributions

Review concept and design and manuscript revision: MY and XL. Electronic and manual literature searches: L-yZ, X-qC, M-xP, and LF. Study selection and data extraction, quality control, and external adviser: L-yZ, X-qC, B-bY, JL, X-jC, and MY. Review analysis: L-yZ, X-qC, and XL. Initial draft writing: L-yZ, X-qC, and B-bY. All authors contributed to the article and approved the submitted version.

## Funding

This study was supported by the National Natural Science Foundation of China (81804152, 82072546, 82074454, and 82174409).

## Conflict of interest

The authors declare that the research was conducted in the absence of any commercial or financial relationships that could be construed as a potential conflict of interest.

## Publisher's note

All claims expressed in this article are solely those of the authors and do not necessarily represent those of their affiliated organizations, or those of the publisher, the editors and the reviewers. Any product that may be evaluated in this article, or claim that may be made by its manufacturer, is not guaranteed or endorsed by the publisher.
