# Supplementary material for: The effect of metformin on ameliorating neurological function deficits and tissue damage in rats following spinal cord injury: A systematic review and network meta-analysis
Source: Front Neurosci. 2022 Aug 11;16:946879. doi: 10.3389/fnins.2022.946879 (PMC9479497; doi:10.3389/fnins.2022.946879)
Supplement: Supplementary file 1 [file Data_Sheet_1.pdf]

The effect of metformin on ameliorating neurological function deficits and tissue damage in rats following spinal cord injury: A systematic review and network meta-analysis

SUPPLEMENTARY MATERIAL

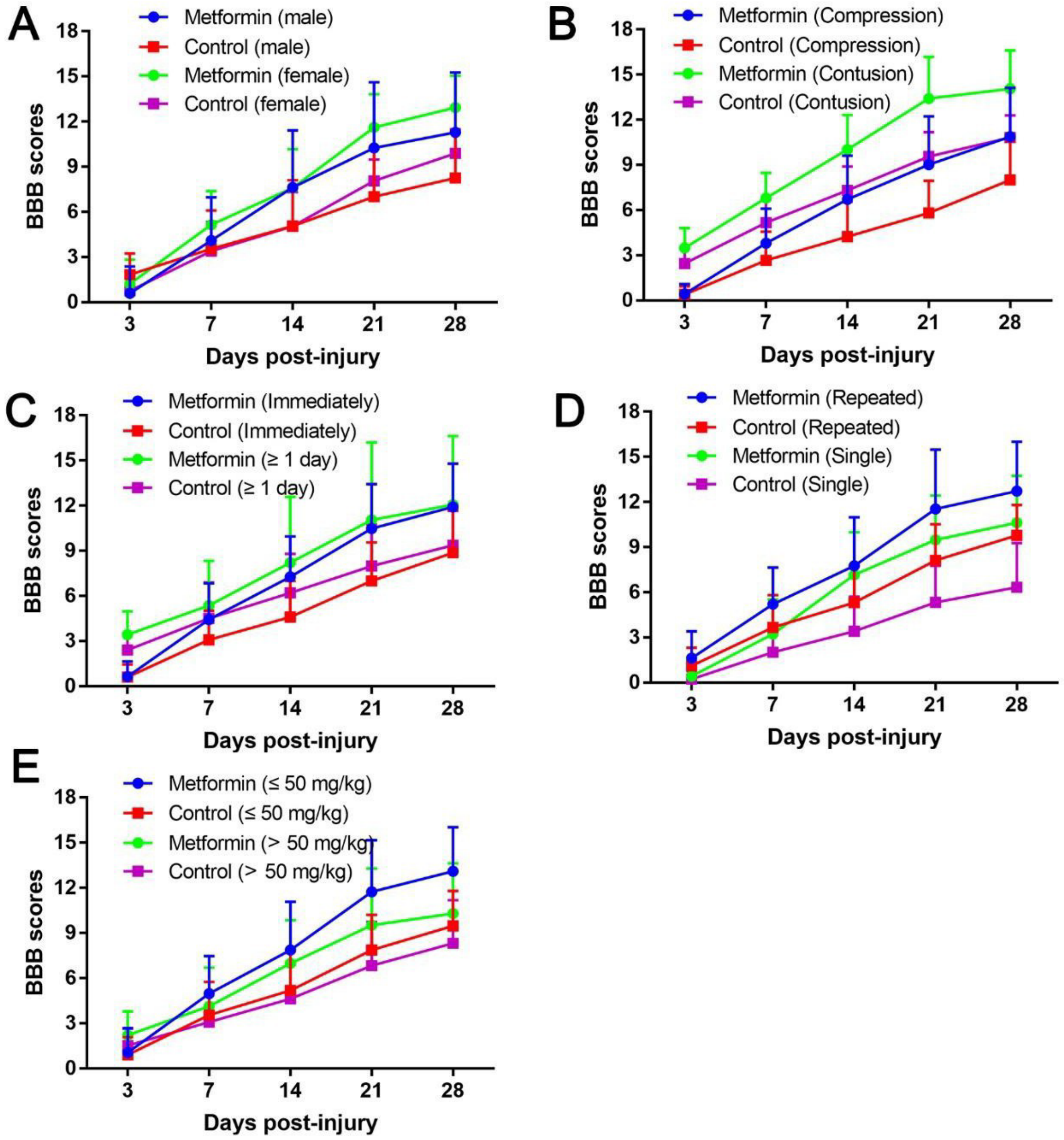

Fig. S1 The pooled BBB scores of metformin and control groups under the different subgroup classifications. (A-E) BBB scores in each subgroup over time.

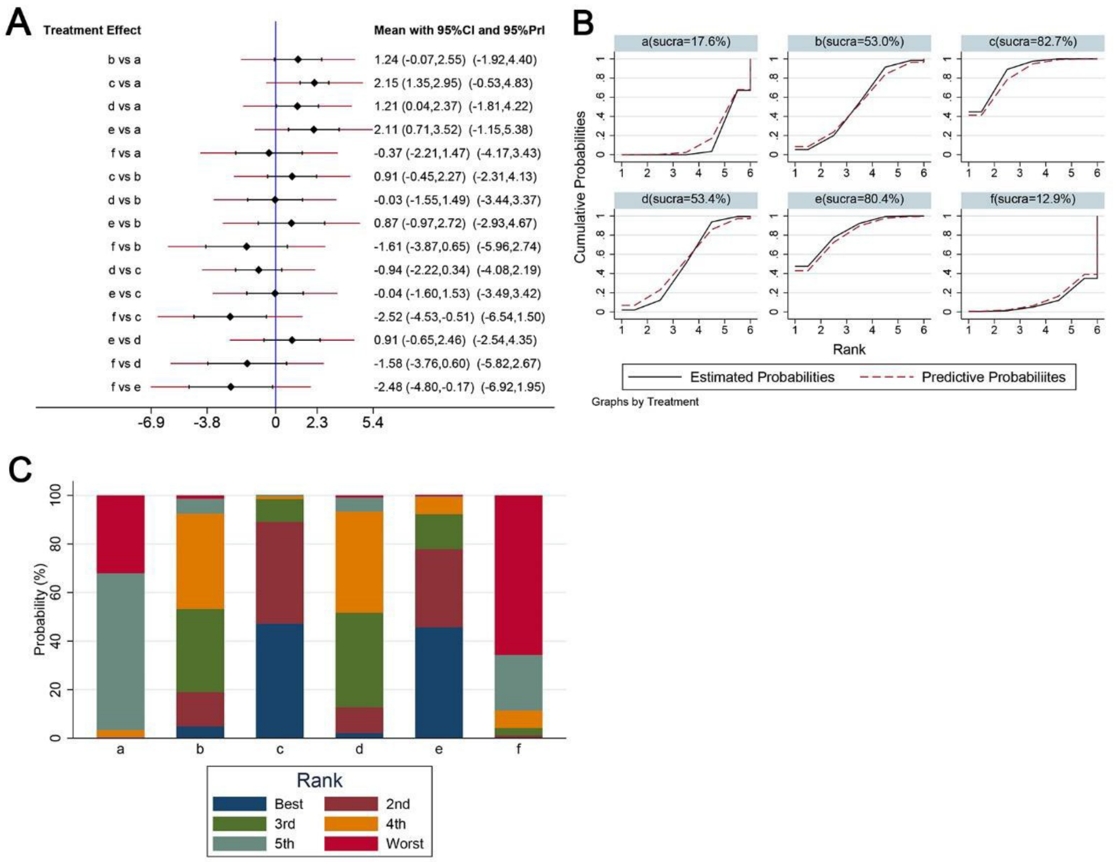

Fig. S2 Network analysis of the effects of metformin at different doses. (A) Forest plot of effect size in different metformin doses according to data on 14th day. (B) SUCRA value ranking of different administration doses according to data on 14th day. (C) histogram of ranking probability of each treatment dose according to data on 14th day.

Table S1. Summary of sensitivity analysis.

BBB scale	Studies exclusion	No. of animals	Weighted mean difference		Heterogeneity	
			95% CI	*P* value	I^2	*P* value
1 BBB scale at 3rd day	All included	164	0.42 [-0.01, 0.85]	0.06	72	0.0004
	No randomized study	66	0.81 [0.01, 1.62]	0.05	74	0.004
	Small sample studies	114	0.58 [-0.25, 1.42]	0.17	84	0.0003
	Afshari K et al. 2018	136	0.47 [-0.05, 0.98]	0.07	75	0.0002
	Guo Y et al. 2018	154	0.36 [-0.07, 0.80]	0.10	74	0.0004
	Wang C et al. 2016	146	0.30 [-0.11, 0.72]	0.16	69	0.002
	Wang HL et al. 2020 1	144	0.42 [-0.01, 0.85]	0.06	72	0.0004
	Wang HL et al. 2020 2	164	0.42 [-0.01, 0.85]	0.06	72	0.0004
	Wu YQ et al. 2021	148	0.53 [0.09, 0.96]	0.02	71	0.001
	Zhang D et al. 2017b 1	154	0.51 [0.02, 0.99]	0.04	70	0.001
	Zhang D et al. 2017b 2	154	0.48 [-0.03, 0.98]	0.07	75	0.0002
	Zhang D et al. 2017c	154	0.46 [-0.01, 0.93]	0.05	75	0.0002
	Zhang T et al. 2020	152	0.25 [-0.12, 0.61]	0.19	53	0.04
	Zhao JW et al. 2021b	154	0.39 [-0.07, 0.86]	0.09	75	0.0002
2 BBB scale at 7th day	All included	198	1.41 [1.01, 1.80]	< 0.00001	52	0.01
	No randomized study	78	1.58 [0.91, 2.25]	< 0.0001	54	0.06
	Small sample studies	138	1.29 [0.68, 1.89]	< 0.0001	69	0.002
	Afshari K et al. 2018	170	1.40 [0.98, 1.81]	< 0.00001	55	0.008
	Guo WD et al. 2019	186	1.27 [0.92, 1.62]	< 0.00001	38	0.08
	Guo Y et al. 2018	188	1.38 [0.98, 1.78]	< 0.00001	54	0.01
	Lin CM et al. 2015	186	1.48 [1.20, 1.75]	< 0.00001	0	0.48
	Wang C et al. 2016	180	1.41 [0.99, 1.83]	< 0.00001	56	0.007
	Wang HL et al. 2020 1	178	1.40 [0.98, 1.82]	< 0.00001	55	0.008
	Wang HL et al. 2020 2	178	1.42 [0.99, 1.85]	< 0.00001	56	0.007
	Wang P et al. 2018	188	1.42 [0.98, 1.87]	< 0.00001	55	0.008
	Wu YQ et al. 2021	182	1.46 [1.04, 1.88]	< 0.00001	55	0.009

	Zhang D et al. 2017b 1	188	1.39 [0.97, 1.81]	< 0.00001	55	0.009
	Zhang D et al. 2017b 2	188	1.40 [0.98, 1.82]	< 0.00001	55	0.008
	Zhang D et al. 2017c	188	1.40 [0.99, 1.80]	< 0.00001	55	0.008
	Zhang T et al. 2020	186	1.48 [1.04, 1.91]	< 0.00001	54	0.01
	Zhao JW et al. 2021b	188	1.40 [0.98, 1.81]	< 0.00001	55	0.008
3 BBB scale at 14th day	All included	186	2.69 [1.92, 3.47]	< 0.00001	82	< 0.00001
	No randomized study	66	2.87 [1.81, 3.93]	< 0.00001	80	0.0004
	Small sample studies	126	2.32 [1.00, 3.64]	0.0006	90	< 0.00001
	Afshari K et al. 2018	158	2.58 [1.78, 3.39]	< 0.00001	83	< 0.00001
	Guo Y et al. 2018	176	2.65 [1.82, 3.47]	< 0.00001	84	< 0.00001
	Lin CM et al. 2015	174	2.96 [2.37, 3.55]	< 0.00001	65	0.0009
	Wang C et al. 2016	168	2.72 [1.88, 3.56]	< 0.00001	84	< 0.00001
	Wang HL et al. 2020 1	166	2.70 [1.88, 3.53]	< 0.00001	84	< 0.00001
	Wang HL et al. 2020 2	166	2.78 [1.95, 3.60]	< 0.00001	83	< 0.00001
	Wang P et al. 2018	176	2.57 [1.76, 3.38]	< 0.00001	83	< 0.00001
	Wu YQ et al. 2021	170	2.79 [1.97, 3.61]	< 0.00001	82	< 0.00001
	Zhang D et al. 2017b 1	176	2.72 [1.87, 3.57]	< 0.00001	84	< 0.00001
	Zhang D et al. 2017b 2	176	2.67 [1.81, 3.52]	< 0.00001	84	< 0.00001
	Zhang D et al. 2017c	176	2.66 [1.85, 3.47]	< 0.00001	84	< 0.00001
	Zhang T et al. 2020	174	2.54 [1.79, 3.29]	< 0.00001	75	< 0.00001
	Zhao JW et al. 2021b	176	2.69 [1.86, 3.52]	< 0.00001	84	< 0.00001
4 BBB scale at 21th day	All included	120	3.89 [2.50, 5.28]	< 0.00001	91	< 0.00001
	No randomized study	50	4.61 [3.21, 6.01]	< 0.00001	77	0.004
	Small sample studies	70	3.06 [0.10, 6.03]	0.04	96	< 0.00001
	Afshari K et al. 2018	92	3.87 [2.38, 5.35]	< 0.00001	93	< 0.00001
	Guo Y et al. 2018	110	3.71 [2.22, 5.19]	< 0.00001	92	< 0.00001
	Lin CM et al. 2015	108	4.43 [3.53, 5.33]	< 0.00001	75	0.0002
	Wang C et al. 2016	102	4.02 [2.48, 5.57]	< 0.00001	92	< 0.00001
	Wang P et al. 2018	110	3.64 [2.14, 5.14]	< 0.00001	92	< 0.00001

	Zhang D et al. 2017b 1	110	3.90 [2.31, 5.49]	< 0.00001	93	< 0.00001
	Zhang D et al. 2017b 2	110	3.99 [2.40, 5.58]	< 0.00001	92	< 0.00001
	Zhang T et al. 2020	108	3.67 [2.18, 5.15]	< 0.00001	90	< 0.00001
	Zhao JW et al. 2021b	110	3.81 [2.30, 5.32]	< 0.00001	93	< 0.00001
5 BBB scale at 28th day	All included	136	3.48 [2.04, 4.92]	< 0.00001	94	< 0.00001
	No randomized study	66	3.71 [2.20, 5.22]	< 0.00001	90	< 0.00001
	Small sample studies	86	3.11 [0.42, 5.79]	0.02	97	< 0.00001
	Afshari K et al. 2018	108	3.35 [1.82, 4.88]	< 0.0001	95	< 0.00001
	Guo Y et al. 2018	126	3.40 [1.86, 4.94]	< 0.0001	95	< 0.00001
	Lin CM et al. 2015	124	3.93 [2.95, 4.90]	< 0.00001	83	< 0.00001
	Wang C et al. 2016	118	3.58 [1.99, 5.17]	< 0.0001	94	< 0.00001
	Wang P et al. 2018	126	3.28 [1.71, 4.85]	< 0.0001	95	< 0.00001
	Wu YQ et al. 2021	120	3.55 [1.98, 5.12]	< 0.0001	95	< 0.00001
	Zhang D et al. 2017b 1	126	3.47 [1.88, 5.05]	< 0.0001	95	< 0.00001
	Zhang D et al. 2017b 2	126	3.51 [1.91, 5.11]	< 0.0001	95	< 0.00001
	Zhang T et al. 2020	124	3.20 [1.93, 4.47]	< 0.00001	87	< 0.00001
	Zhao JW et al. 2021b	126	3.54 [1.93, 5.15]	< 0.0001	94	< 0.00001
