# Supplementary material for: The effect of metformin on ameliorating neurological function deficits and tissue damage in rats following spinal cord injury: A systematic review and network meta-analysis
Source: Front Neurosci. 2022 Aug 11;16:946879. doi: 10.3389/fnins.2022.946879 (PMC9479497; doi:10.3389/fnins.2022.946879)
Supplement: Supplementary file 2 [file Data_Sheet_2.docx]

**Appendix 1: PubMed database search strategy**

**Search Strategy**

(metformin[MeSH Terms] OR biguanides[MeSH Terms] OR guanidines[MeSH Terms] OR dimethylbiguanidine[All Fields] OR dimethylguanylguanidine[All Fields] OR glucophage[All Fields] OR Metformin Hydrochloride[All Fields] OR Hydrochloride, Metformin[All Fields] OR Metformin HCl[All Fields] OR HCl, Metformin[All Fields] OR metformin[All Fields] OR biguanide[All Fields] OR guanidine[All Fields] OR antidiabetics[All Fields] OR antidiabetic[All Fields] OR AMP-activated protein kinase agonist[All Fields] OR AMPK agonist[All Fields]) AND (spinal cord injuries[MeSH Terms] OR trauma, nervous system[MeSH Terms] OR spinal cord diseases[MeSH Terms] OR central cord syndrome[MeSH Terms] OR spinal cord compression[MeSH Terms] OR spinal cord trauma[All Fields] OR cord trauma, spinal[All Fields] OR cord traumas, spinal[All Fields] OR trauma, spinal cord[All Fields] OR traumas, spinal cord[All Fields] OR spinal cord traumas[All Fields] OR myelopathy, traumatic[All Fields] OR myelopathies, traumatic[All Fields] OR traumatic myelopathies[All Fields] OR traumatic myelopathy[All Fields] OR spinal cord injury[All Fields] OR cord injury, spinal[All Fields] OR cord injuries, spinal[All Fields] OR injury, spinal cord[All Fields] OR injuries, spinal cord[All Fields] OR spinal cord injuries[All Fields] OR spinal cord transection[All Fields] OR cord transection, spinal[All Fields] OR cord transections, spinal[All Fields] OR transection, spinal cord[All Fields] OR transections, spinal cord[All Fields] OR spinal cord transections[All Fields] OR spinal cord laceration[All Fields] OR cord laceration, spinal[All Fields] OR cord lacerations, spinal[All Fields] OR laceration, spinal cord[All Fields] OR lacerations, spinal cord[All Fields] OR spinal cord lacerations[All Fields] OR spinal cord contusion[All Fields] OR cord contusion, spinal[All Fields] OR cord contusions, spinal[All Fields] OR contusion, spinal cord[All Fields] OR contusions, spinal cord[All Fields] OR spinal cord contusions[All Fields] OR spinal cord compression[All Fields] OR cord compression, spinal[All Fields] OR cord compressions, spinal[All Fields] OR compression, spinal cord[All Fields] OR compressions, spinal cord[All Fields] OR spinal cord compressions[All Fields] OR spinal cord hemisection[All Fields] OR cord hemisection, spinal[All Fields] OR cord hemisections, spinal[All Fields] OR hemisection, spinal cord[All Fields] OR hemisections, spinal cord[All Fields] OR spinal cord hemisections[All Fields] OR spinal cord ischemia[All Fields] OR cord ischemia, spinal[All Fields] OR ischemia, spinal cord[All Fields] OR dorsal column injury[All Fields] OR corticospinal tract injury[All Fields]) AND (rats[MeSH Terms] OR murinae[MeSH Terms] OR rats, inbred strains[MeSH Terms] OR rats[All Fields] OR rat[All Fields] OR rattus[All Fields] OR rats, laboratory[All Fields] OR rat, laboratory[All Fields] OR laboratory rats[All Fields] OR laboratory rat[All Fields])
